# Switching, fast and slow: Deciphering the dynamics of memory search, its brain connectivity patterns, and its role in creativity

**DOI:** 10.1162/IMAG.a.1018

**Published:** 2025-11-18

**Authors:** Marcela Ovando-Tellez, Lucie Vigreux, Yoed N. Kenett, Mathias Benedek, Thomas T. Hills, Benoit Beranger, Alizée Lopez-Persem, Victor Altmayer, Theophile Bieth, Emmanuelle Volle

**Affiliations:** Sorbonne University, FrontLab at Paris Brain Institute (ICM), INSERM, CNRS, Paris, France; Brain Connectivity and Behaviour Laboratory, Paris, France; Groupe d’imaginerie fonctionelle (GIN), Institut des maladies Neurodegeneratives (IMN)—UMR 5293, CNRS, Bordeaux, France; Faculty of Data and Decision Sciences, Technion—Israel Institute of Technology, Haifa, Israel; Institute of Psychology, University of Graz, Graz, Austria; Department of Psychology, University of Warwick, Coventry, United Kingdom; Sorbonne University, CENIR at Paris Brain Institute (ICM), INSERM, CNRS, Paris, France; Neurology department, Pitié-Salpêtrière Hospital, AP-HP, Paris, France

**Keywords:** creativity, semantic foraging, fluency, executive control, semantic memory, functional connectivity

## Abstract

Creative ideas emerge from the process of searching and combining concepts in memory, involving both associative and controlled mechanisms. How these processes unfold during memory search and relate to creativity remains unclear. We explored the neurocognitive underpinnings of semantic memory search using a clustering–switching framework and the Marginal Value Theorem (MVT) from optimal foraging theory. During an associative fluency task with polysemous words, most responses aligned with MVT predictions, but some deviated from them. These behavioral results were replicated in an independent sample. Connectome-based modeling revealed that functional brain connectivity predicted these MVT-deviant patterns and mediated the relationship between brain connectivity and creative performance. These findings suggest that the cognitive policy favoring creativity may differ from the policy optimizing fluency (i.e., MVT). This study introduces novel measures of semantic search, identifies their neurocognitive correlates, and underscores the importance of search patterns in understanding creative abilities.

## 1 Introduction

Generating creative ideas involves searching, reorganizing, and combining concepts in memory in novel and useful ways (Benedek et al., 2023; Kenett, 2023). Creativity is supported by two key cognitive processes: associative and controlled processes (Benedek & Jauk, 2018; Benedek et al., 2023; Volle, 2018). Associative processes have been explored since the 60s, inspired by Mednick’s theory of creativity (Beaty & Kenett, 2023; Mednick, 1962). This theory hypothesizes that creativity depends on the organization of the concepts in memory, that is, memory structure (He et al., 2021), allowing more creative individuals to easily connect unrelated associative elements in memory compared with less creative individuals. Consistent with this theory, supporting evidence shows that more creative individuals have better associative abilities, more uncommon word associations, and a more flexible organization of semantic associations in memory (Beaty & Kenett, 2023; Beaty et al., 2014; Bendetowicz et al., 2018; Benedek, Könen, et al., 2012; Kenett & Faust, 2019; Marron et al., 2018; Ovando-Tellez et al., 2023). While associative processes can allow remote connections, producing creative ideas also involves controlled processes that enable efficient retrieval and navigation within memory (Beaty et al., 2014; Benedek & Jauk, 2018; Benedek et al., 2023; Volle, 2018) and the inhibition of common and unoriginal ideas (Beaty et al., 2017; Camarda et al., 2018; Lloyd-Cox et al., 2021). The role of controlled processes has been suggested by the correlation between executive function abilities and creative abilities (Benedek & Fink, 2019; Beaty et al., 2017; Chrysikou, 2019; Edl et al., 2014; Jauk et al., 2013; Lee & Therriault, 2013). For instance, better performance in divergent thinking tasks, which involve open-ended multidirectional memory search, has been associated with inhibition (Benedek, Franz, et al., 2012; Benedek et al., 2014; Camarda et al., 2018; Krumm et al., 2018), task shifting (Krumm et al., 2018; Pan & Yu, 2018), and broad retrieval abilities (Benedek, Franz, et al., 2012; Benedek et al., 2017; Forthmann et al., 2019; Miroshnik et al., 2023; Silvia et al., 2013). The latter involves retrieving information from long-term memory fluently and flexibly (Schneider & McGrew, 2018), which is fundamental for creative thinking (Benedek, Franz, et al., 2012; Forthmann et al., 2019). In addition to divergent thinking, controlled processes have also been related to the remote associate task (Lee & Therriault, 2013; Mednick, 1962; Smith et al., 2013; Volle, 2018), which involves a multidirectional but multiconstrained memory search (Davelaar, 2015) that converges to a unique solution. Hence, existing research indicates a critical role of both associative and controlled processes in creative ideation. However, the dynamic interaction between these processes that promotes creativity during memory search remains unclear.

Beyond creativity research, the interplay between associative and controlled processes during memory search has been explored using classical fluency tasks, where participants are asked to retrieve as many members of a category (e.g., animals) as possible within a limited time. Previous studies (Troyer et al., 1997, 1998) have categorized the responses in these tasks as clustering responses, referring to successive responses within the same subcategory (e.g., pets or wild animals), and switching ones, referring to moving to another category. According to Troyer’s research, clustering reflects memory representations and associations, supported by temporal brain regions, while switching involves controlled processes supported by frontal brain regions (Troyer et al., 1997). Importantly, clustering and switching responses have also been observed in divergent thinking tasks (Acar & Runco, 2017; Hass, 2017; Mastria et al., 2021; Nusbaum & Silvia, 2011). In this context, switching was related to controlled processes (Nusbaum & Silvia, 2011), longer latencies (Acar & Runco, 2017), and larger investment of cognitive resources at the brain level (Mastria et al., 2021).

In a previous study, we extended this framework by developing the Polysemous Fluency Task (PolyFT) (Ovando-Tellez, Benedek, et al., 2022), using polysemous words as cues to characterize behavioral components of clustering and switching. Findings from Ovando-Tellez, Benedek, et al. (2022) indicated that clustering and switching components both relate to individual differences in creative abilities but reflect distinct neurocognitive processes. Specifically, the clustering component was linked to greater fluency and originality in a divergent thinking task and involved higher brain functional connectivity between executive control and attentional networks. In contrast, the switching component was related to combining remote associates (measured by the remote associate task), semantic memory structure, and executive abilities, and involved brain interactions between the default mode and executive control networks. These results were not entirely consistent with the predictions from Troyer’s hypothesis (Troyer et al., 1997). Specifically, our clustering component, expected to rely on memory associations, was predicted by brain networks involved in executive control and attention. Our switching component was predicted not only by networks involved in executive control, consistent with Troyer’s hypothesis, but also by the default mode network, which is assumed to support associative thinking. We interpreted these findings as indications that switching involves the interplay between associative and controlled processes for a flexible search (Bendetowicz et al., 2018; Ovando-Tellez et al., 2019), whereas clustering involves attentional control processes to maximally exploit a cluster, aligning with the persistent pathway of the dual pathway to creativity model (Boot et al., 2017; Nijstad et al., 2010). According to this model, creative performance can be achieved via a persistence pathway, which relies on focused and systematic thinking and prolonged search processes, or a flexibility pathway that includes a broad attentional scope, facilitated access to remote semantic concepts, and flexible switching between perspectives.

The partial inconsistencies between our results (Ovando-Tellez, Benedek, et al., 2022) and those of previous studies (Troyer et al., 1997, 1998) prompt the need for further exploration into the processes that occur during memory search related to creativity. To address this gap, we conducted the current study where we re-analyzed data from Ovando-Tellez, Benedek, et al. (2022) using the marginal value theorem (MVT) (Charnov, 1976; Hills et al., 2012, 2015) that describes memory search as a trade-off between exploitation of a given cluster and exploration of different clusters (Hills et al., 2012, 2015; Todd & Hills, 2020). Hills et al. (2012) have shown that memory search parallels the optimal foraging of animals in space when searching for food (Charnov, 1976). In line with the MVT of optimal foraging (Charnov, 1976), Hills et al. (2012) demonstrated that, during a semantic fluency task, switching to a different cluster (i.e., a subcategory in the fluency task) occurs when the retrieval rate within the current cluster falls below a threshold. These switches take longer (longer inter-response times—IRT) than staying within the same cluster. In addition, participants optimally leave a cluster when the current retrieval rate (captured by the current IRT) reaches their marginal value, computed individually as the average IRT over the whole task (hereafter referred to as long-term IRT). By incorporating the MVT framework into our clustering–switching analysis of the PolyFT, we aimed to clarify our previous interpretations (Ovando-Tellez, Benedek, et al., 2022) and provide a more comprehensive understanding of how clustering and switching responses reflect different neurocognitive processes involved in the trade-off between exploiting clusters and exploring new clusters during memory search, and their relationship with creative abilities. In addition to reanalyzing this previous study in light of the MVT, we collected and analyzed a new dataset to ensure the replicability of our findings. Based on previous findings in memory search, we formed four hypotheses. While our first hypothesis was based on theoretical background, the subsequent hypotheses were integrated based on the results of the analyses testing the first hypothesis.

First, we hypothesized that memory search patterns during the PolyFT would be consistent with the predictions of the MVT for semantic memory (Hills et al., 2012), allowing us to characterize switching responses according to MVT principles (see also Ackerman, 2014). We classified the responses produced by the participants during the PolyFT as clustering (successive responses referring to the same meaning) and switching (transition to a different meaning) and analyzed their IRTs (Hills et al., 2012, 2015; Todd & Hills, 2020). Consistent with optimal foraging in semantic memory, we expected participants to leave a cluster (i.e., to switch to a new cluster) when the current retrieval rate approached their marginal value, computed individually as the long-term IRT, and that, in line with MVT policies, the closer participants are to this value before switching, the more efficient their retrieval would be. While our analyses confirmed that search behavior in general followed the MVT, with clustering responses occurring faster and switching responses occurring slower than the marginal value, they also revealed clustering responses occurring slower and switching responses occurring faster than predicted by this model. To capture this variability, we categorized responses into four novel types: fast clustering and slow switching responses (both consistent with the MVT prediction), and fast switching and slow clustering responses (unexpected based on MVT). We interpreted slow clustering and fast switching responses as potential over-exploitation and over-exploration, respectively, as they deviate from the optimal foraging expectation. Exploratory analyses then retested the MVT predictions separately for fast and slow switching responses.

Second, we assumed that this finer characterization of search patterns may help to clarify the unexpected results in Ovando-Tellez, Benedek, et al. (2022), mentioned above, which suggested that both clustering and switching involve controlled processes. We hypothesized that slow responses may reflect the involvement of controlled processes, whereas fast responses reflect spontaneous associative processes dependent on semantic memory. Accordingly, slow switching would align with Troyer’s findings (Troyer et al., 1997), while slow clustering would reflect attentional controlled processes to exploit a cluster. Conversely, fast switching may reflect spontaneous flexibility and fast clustering associative processes of memory retrieval in agreement with Troyer’s findings (Troyer et al., 1997). Thus, according to hypothesis 2, the number of slow clustering and slow switching responses (but not their fast counterparts) should relate to executive function abilities.

Third, we hypothesized that memory search components related to creative abilities can be predicted by brain functional connectivity. Previous studies using connectome-based predictive modeling (CPM) have shown that individual patterns of brain functional connectivity can predict individual differences in creativity (Beaty et al., 2018; Ovando-Tellez, Benedek, et al., 2022; Ovando-Tellez, Kenett, et al., 2022). Here, we used a CPM approach to predict memory search components from individual brain functional connectivity patterns, as in Ovando-Tellez, Benedek, et al. (2022), but with a finer characterization of the components distinguishing the four types of responses: slow switching, fast switching, slow clustering, and fast clustering. We predicted the number of fast switching and slow clustering responses (MVT-deviant patterns) from brain connectivity, but predictions for slow switching and fast clustering responses (patterns consistent with Troyer’s hypothesis and the MVT) were not significant.

Finally, we examined the relevance of brain connectivity patterns predictive of these patterns of memory search to creativity using mediation analyses. In our prior study (Ovando-Tellez, Benedek, et al., 2022), clustering correlated with divergent thinking tests and switching correlated with the remote associate task, and they were interpreted as reflecting the persistence and flexibility pathways for creativity (Nijstad et al., 2010), respectively. We thus expected to observe a link between the number of fast switching responses and the performance on the remote associate test, and between the number of slow clustering responses and divergent thinking measures. We interpreted the relationships between fast switching and slow clustering and creativity in light of the flexibility and persistence pathway of the dual pathway to creativity model (Nijstad et al., 2010). Details of all hypotheses tested in this study are summarized in Table 1.

**Table 1.** Summary of hypotheses.

Hypothesis	Variables	Statistical test	RESULTS	Figure/Table
**Hypothesis 1: General memory search patterns during the PolyFT are consistent with the MVT**				
Participants leave a cluster when they reach their *long-term IRT*	*Clustering_IRTr (positions -2, -1, +1, +2)*	Wilcoxon test	Confirmed	Figure 2A, B
	*Switching_IRTr*			
Switching responses occurring closer to reaching the *long-term IRT* yield more responses	*PolyFT_fluency*	Linear regression	Confirmed	Figure 2C, D
	*pre-switch IRT (position -1)*			
	*long-term IRT*			
Responses with longer *IRT* are more semantically dissimilar	*IRT*	Mixed regression model	Confirmed	Figure 3
	*IRS*			
Some responses were inconsistent with the MVT (clustering responses occurring slower and switching responses occurring faster than predicted by the MVT)	*Clustering_IRTr*	Comparison to marginal value	Data-driven observation	Figure 3, Table 4
	*Switching_IRTr*			
Fast switching and slow switching responses involve distinct mechanisms	*Clustering_IRTr (positions -2, -1, +1, +2)*	Wilcoxon test	Confirmed	Figure 4
	*Fast/Slow Switching_IRTr*			
**Hypothesis 2: Clustering and switching involve controlled processes, with these processes captured by slow responses**				
*Slow-Clustering* and *Slow-Switching* relate to executive functions	*Fast-Clustering Slow-ClusteringFast-Switching Slow-Switching*	Spearman correlations	Partially confirmed	Table S7
	Executive function scores			
**Hypothesis 3: Memory search components can be predicted by brain functional connectivity**				
Individual patterns of brain functional connectivity predict *Fast-Clustering, Fast-Switching, Slow-Clustering,* and *Slow-Switching*	*Fast-Clustering Slow-ClusteringFast-Switching Slow-Switching*	CPM – Spearman correlations	Confirmed only for *Slow-Clustering* and *Fast-Switching*	Figure 5
	Functional connectivity matrices			
**Hypothesis 4: Brain connectivity patterns predictive of memory search patterns are relevant for creative abilities**				
Memory search patterns mediate the brain–creativity relationship	*Fast-Clustering Slow-ClusteringFast-Switching Slow-Switching*	Mediation analysis	Confirmed	Figure 6
	Functional connectivity matrices			
	Creativity scores			

This table outlines the four hypotheses tested in the study, specifying the corresponding variables analyzed, statistical tests, results, and the figures or tables where the results are presented.

## 2 Material and Methods

### 2.1 Study 1

#### 2.1.1 Participants

We reanalyzed the sample of 86 participants (43 women, mean age = 25.5 years, SD = 3.48 years) collected by Ovando-Tellez, Benedek, et al. (2022) who performed the PolyFT, tests of executive control functions, a battery of creativity tasks, and underwent a brain MRI. All participants were French native speakers, right handed, with normal or corrected-to-normal vision and no neurological disorder, cognitive disability, or medication affecting the central nervous system. Participants gave informed written consent and received monetary compensation for their participation. The French ethics committee “CPP Sud Mediterranee IV” approved the study and all methods were performed in accordance with the relevant guidelines and regulations.

#### 2.1.2 General procedure

**Fig. 1.**
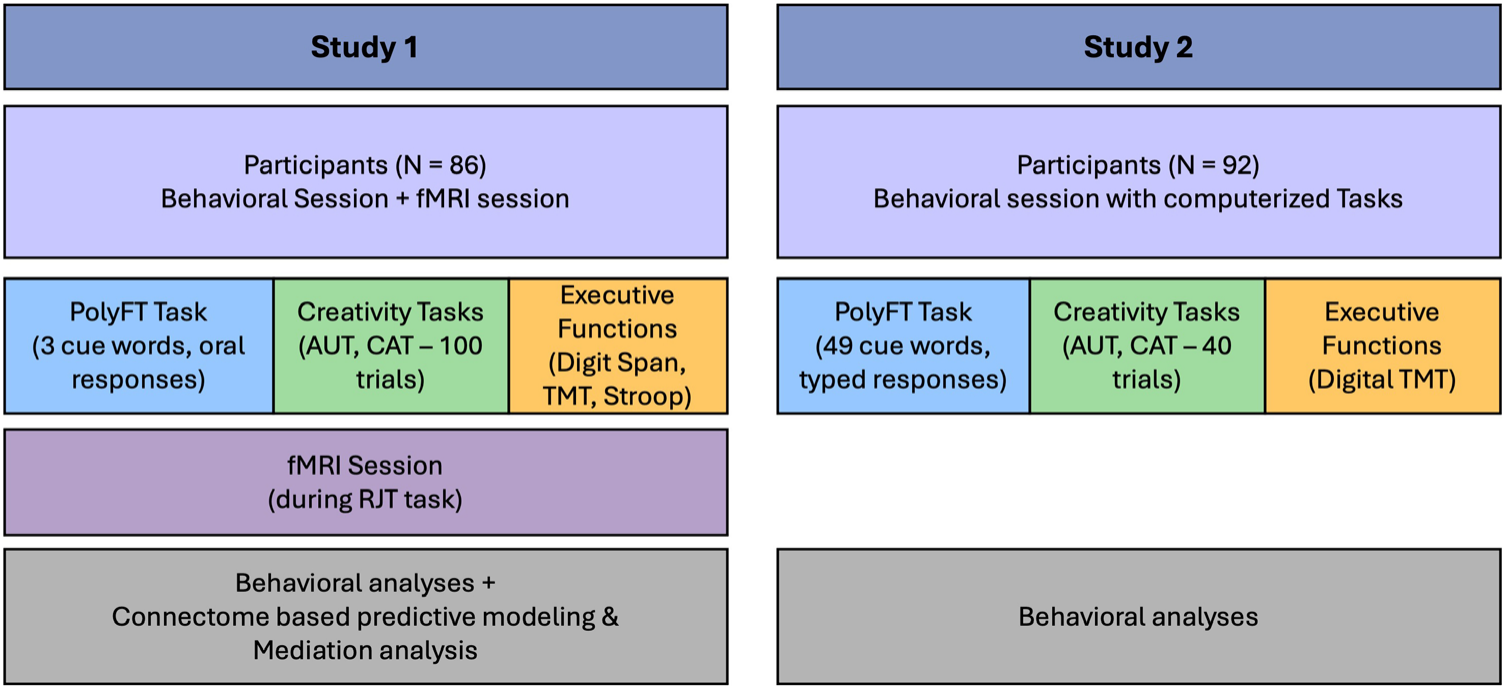
Overview of the experimental design and tasks used in Study 1 and Study 2. This diagram illustrates the design and methods used in both studies.

Participants first completed an MRI scan session during which they performed a task of relatedness judgments (see Supplementary Methods S1 in Supplementary Material), followed by a rest session. Then, outside the scanner, participants completed the PolyFT and a set of creativity and executive function tasks. The experimental design and all variables used in this study are summarized in Figure 1 and Table 2.

**Table 2.** Summary of the tasks and variables used in Study 1 and Study 2.

Task	Task details	Variable	Description
Memory search			
Polysemous word fluency task (PolyFT)	Participants generated as many single-word associations as possible for ambiguous cue words (1 minute in Study 1; 1.5 minutes in Study 2). Responses were categorized as clustering (same meaning as previous response) or switching (different meaning)	*PolyFT_fluency*	Mean number of unique responses across responses and cue words
		Inter-Response Time (*IRT*)*	Time between successive responses
		Inter-Response Similarity (*IRS*)*	Word2vec cosine similarity between successive responses
		long-term inter-response time (*long-term IRT*)	Participant’s mean *IRT* across all responses and cue words. Serves as reference value for *IRTr*
		Long-term inter-response similarity (*long-term IRS*)	Participant’s mean *IRS* across all responses and cue words. Serves as reference value for *IRSr*
		Inter-Response Time ratio (*IRTr*)*	Ratio of each *IRT* to the individuals’ *long-term IRT* for that cue word
		Inter-Response Similarity ratio (*IRSr*)*	Ratio of each *IRS* to the individuals’ *long-term IRS* for that cue word
		*pre switch IRT*	Mean *IRT* for clustering responses occurring immediately before a switch
		*Clustering_IRT*	Mean *IRT* for responses classified as clustering.
		*Switching_IRT*	Mean *IRT* for responses classified as switching
		*Clustering_IRTr*	Mean *IRTr* for responses classified as clustering
		*Switching_IRTr*	Mean *IRTr* for responses classified as switching
		*Fast-Clustering Slow-Clustering*	Number of clustering responses with *IRTr* < 1 (fast) or *IRTr* > 1 (slow)
		*Fast-Switching Slow-Switching*	Number of switching responses with *IRTr* < 1 (fast) or *IRTr* > 1 (slow)
Creative abilities (see Supplementary Methods S3)			
Alternative Uses Task (AUT)	Participants generated alternative uses for three objects (3 minutes per object)	*AUT-fluency*	Total number of alternative uses generated across the three objects.
		*AUT-uniqueness*	Total number of ideas produced by fewer than 5% of the participants across the three objects.
		*AUT-ratings*	Top-2 ideas per object rated for creativity (0–4) by 5 external raters; scores averaged across raters and objects
Combination of Associates Task (CAT)	Participants solved 100 trials (40 trials in Study 2) of cue-word triplets within 30 s each, reporting if solutions came with insight (Eureka). Trials varied in semantic distance (50 close, 50 distant)	*CAT_CR*	Accuracy in performance calculated as the % of correct responses across all trials.
		*CAT_index*	Relative performance in distant versus close trials (difference divided by overall accuracy)
		*CAT_eureka*	% of correct trials reported with insight (Eureka)
Executive functions (see Supplementary Methods S4)			
Digit span test	Participants repeated 16 increasing strings of numbers in reverse order.	*Backward-span*	Sum of correct responses (higher = better working memory ability)
Trail Making Test (TMT)	Connect numbers (Part A) or alternate numbers and letters (Part B) as fast as possible.	*TMT-shifting*	Set-shifting assessed by the time difference between part B and part A (higher = lower set-shifting ability)
Stroop test	Name ink colors of 100 color words with and without interference.	*Stroop-interference*	Time difference between interference and color-naming conditions (higher = lower inhibition).

For the PolyFT, variables were calculated at the individual response level, and then across all responses and cue words. All PolyFT variables represent the mean of the measures computed across responses and cue words, except for those marked with a star (*), which were computed at the level of each individual response. Measures for the creative and executive function abilities are described with their respective tasks and variables.

#### 2.1.3 The polysemous word-fluency task (PolyFT)

##### 2.1.3.1 Task description

In the PolyFT, participants were presented with a cue word and were instructed to freely generate all the single-word associations they could think of in relation to the given cue word for 1 minute (Ovando-Tellez, Benedek, et al., 2022). In total, we presented three cue words that were ambiguous French polysemous words: SOMME, GLACE, and RAYON. All the cue words have high lexical frequency (>20 occurrences per million in a large corpus according to the French lexicon project, http://www.lexique.org/) and at least five different meanings according to the French dictionary and the French linguistic resource for research (Centre National de Ressources Textuelles et Lexicales; https://www.cnrtl.fr/). Details on the instructions and the different meanings of the cue words are provided with an English translation in Supplementary Methods S2 and Supplementary Table S1. The cue words were presented visually on a paper sheet and read aloud by the experimenter. Participants gave their responses aloud, which were audio recorded and written down on a computer by the experimenter. We manually cleaned the responses for typing errors and homogenized the plural and grammatical gender. We quantified the total number of unique responses given by the participants for the three cue words and used the mean as the *PolyFT_fluency* score.

##### 2.1.3.2 Categorization of the PolyFT responses into clustering and switching

We categorized each produced response as belonging to one of the meanings of each cue word. Using this classification, we identified clustering responses as those referring to the same meaning than the previous response, and switching responses as those referring to a different meaning than the previous one.

#### 2.1.4 Exploration of optimal foraging in semantic memory using the marginal value theorem

##### 2.1.4.1 The inter-response time of retrieved words within and between clusters

In addition to the clustering and switching responses categorization, we analyzed the inter-response time (*IRT*) of the participants’ responses during the PolyFT. To compute the *IRT*, we used the software Audacity 3.1 (https://www.audacityteam.org), manually marking the initial and final time of each response. We then calculated the *IRT*s for all consecutive responses generated by the participants as the difference between the end of a given response and the beginning of the next one. For each participant and each cue word, we computed the *long-term IRT* as the mean *IRT* across responses.

##### 2.1.4.2 The inter-response semantic similarity of retrieved words within and between clusters

We explored the inter-response semantic similarity (*IRS*) using pre-trained Word2vec models for French. Word2vec is a word embedding algorithm based on neural networks. It learns vector representations of the words in a text so that words that share similar contexts are represented by close numerical vectors (Mikolov et al., 2013). We downloaded a pre-trained Word2vec model for French from https://fauconnier.github.io/ (Fauconnier, 2015). This model was built from a 1.6 billion-word corpus constructed from websites with .fr domains, as described in Baroni et al. (2009). For each word, we extracted the word embeddings using Word2vec, then computed the cosine similarity for each pair of consecutive words. Values between -1 (low similarity) and 1 (high similarity) indicate the semantic similarity between two words.

##### 2.1.4.3 Consistency of semantic retrieval in PolyFT with the marginal value theorem

To determine whether memory search patterns during the PolyFT are consistent with the MVT for semantic memory (Hills et al., 2012), we first explored whether the marginal value separates clustering and switching responses as predicted by the MVT (details of all hypotheses tested in this study are summarized in Table 1). Specifically, we examined whether participants leave a cluster when they reach their *long-term IRT* that corresponds to the marginal value (Hills et al., 2012, 2015).

For each response and each cue word, we computed the *IRTr* as the ratio between *IRT*s and individuals’ *long-term IRT* for that cue word. An *IRTr* value of 1 indicates that the *IRT* of a given response corresponds to the individual’s *long-term IRT* for the cue word. Values above 1 indicate a longer *IRT* than the *long-term IRT*, while values below 1 indicate a shorter *IRT*.

We then computed and compared the mean *IRTr* for switching responses (*Switching_IRTr*) and clustering (*Clustering_IRTr*) responses. Additionally, we analyzed the *Clustering_IRTr* values for each position relative to a switching response (i.e., positions -2, -1, +1, +2). We identified the responses preceding and following the switches as follows: Responses in positions -2 and -1 are the second last and last responses within the same cluster before the switch, respectively. The switching response (S) is the first response in a new cluster, and responses in positions +1 and +2 are the first and second responses within the new cluster after the switching response, respectively. Additional analyses explored the dynamics of *IRT*s before a switch.

We then explored whether participants who switch when reaching their *long-term IRT* are more optimal, meaning they generate more responses during the task than participants who switch before or after reaching their *long-term IRT*. Following the optimal foraging approach, an early switch might imply under-exploitation, while a late switch might imply over-exploitation and under-exploration of the clusters. We calculated the absolute difference between the mean *IRT* for clustering responses before switching responses (i.e., responses in position -1, preceding the switch; *pre-switch IRT*) and the *long-term IRT*. We regressed this value on the number of total responses produced during the task.

Finally, applying the same method as described for *IRTr*, we explored whether we observe a similar pattern for *IRSr* but in the opposite direction, wherein participants reach their average semantic retrieval (i.e., *long-term IRS*) during clustering responses, whereas in switching responses, they retrieve more dissimilar or distant concepts than the average.

The computations of *Clustering_IRTr* and *Switching_IRTr* were conducted first for each response and then averaged across the three cue words and participants. All analyses and plots were performed with MATLAB (R2021b, The MathWorks, Inc., USA) and R studio Version 2022.07.1.

#### 2.1.5 Variability in adherence to the marginal value theorem: Fast and slow switching and clustering processes

Based on the MVT, clustering responses are expected to occur before participants reach their *long-term IRT*, while switching responses are expected to occur after they reach it. However, as not all responses align with these expectations, we characterized PolyFT responses generated by participants in relation to their *IRTr*. Responses with *IRTr* values lower than 1 were responses with *IRT*s below the individual’s *long-term IRT* and were classified as “fast.” Conversely, responses with *IRTr* values higher than 1 were responses with *IRT*s exceeding the individual’s *long-term IRT* and were classified as “slow.” Combined with the clustering and switching coding, *IRT*s allowed us to categorize responses into four types: fast clustering and fast switching responses, and slow clustering, and slow switching responses. We then quantified the number of responses generated by each participant for each of these four response types (*Fast-Clustering, Fast-Switching, Slow-Clustering, Slow-Switching*), as well as their mean *IRT*. *Slow-Clustering, Fast-Clustering, Slow-Switching*, and *Fast-Switching* thus refer to the variables measuring the number of responses of each type.

#### 2.1.6 Creativity tasks

Details on the task instructions and scoring are provided in Supplementary Methods S3 of the Supplementary Material.

##### 2.1.6.1 Alternative uses task (AUT)

In the AUT, participants were instructed to generate as many alternative uses as possible for three common objects: tire, bottle, and knife (Guilford, 1967). The experimenter presented the instructions orally, and then participants read them on the computer screen before starting the task. After reading the instructions, the object’s name was displayed on the computer screen and remained until the time ran out. Participants were given 3 minutes for each object to write all their responses on the computer using a keyboard. At the end of the 3 minutes, participants were asked to select their two most creative ideas for each object (top-2) (Benedek et al., 2013; Silvia et al., 2008). We computed three scores for each object: *AUT_fluency*, *AUT_uniqueness*, and *AUT_ratings* (see Table 2).

##### 2.1.6.2 The combination of associates task (CAT)

The CAT is an adaptation of the classical creativity task named the Remote Associates Test (Mednick, 1962). In the CAT, participants were presented with 100 trials composed of triplets of cue words that, at first glance, seem unrelated. For each trial, participants were asked to find a fourth word (i.e., the word solution) that relates to all the cue words within 30 seconds. In this version of the task, the trials vary on the semantic distance between the cue words and the solution, as described in previous articles (Bendetowicz et al., 2017, 2018; Ovando-Tellez et al., 2023) being composed by 50 close trials (low semantic distance between the cue words and the response) and 50 distant trials (high semantic distance between the cue words and the response). At the end of each trial, participants were asked to report whether the solution was found with a feeling of insight or Eureka, that is, when the solution comes to mind suddenly and effortlessly (Kounios & Beeman, 2014; Topolinski & Reber, 2010). We computed three scores: *CAT_CR*, *CAT_index*, and *CAT_eureka* (see Table 2).

#### 2.1.7 Executive function tests

We assessed executive functions with three classical neuropsychological tests: digit span (working memory), trail-making (set-shifting), and Stroop (inhibition). Below, we provide a brief description of each task and the performance measures. Further details are provided in Table 2 and Supplementary Methods S4.

##### 2.1.7.1 Digit span test

We used the digit span test of the Wechsler Adult Intelligence Scale (WAIS) (Wechsler, 2008) to assess working memory abilities. We used the digit backward-span test’s performance criteria as outlined in the WAIS IV manual. Participants were presented with sequences of numbers of increasing length and were asked to repeat them aloud in reverse order. Performance was quantified as the total number of correctly recalled sequences (*Backward-span*).

##### 2.1.7.2 Trail-making test

The trail-making test assesses set-shifting (Reitan, 1992). In this task, participants connected numbers (Part A) or alternated numbers and letters (Part B). We recorded the time participants took to complete each part of the task. We quantified the difference between the time to complete the second minus the first part as *TMT-shifting*.

##### 2.1.7.3 Stroop test

The Stroop test (Stroop, 1935) was used to measure inhibition. We used the French version (Chatelois et al., 1993), in which participants named ink colors of words with and without interference. We recorded the time participants took to complete each part of the task. We quantified the interference effect (*Stroop-interference*) as the difference in time to complete ink naming with interference and without interference.

#### 2.1.8 Statistical analysis

To explore whether the semantic retrieval patterns during the PolyFT are consistent with the MVT, we ran paired Wilcoxon tests to compare *Clustering_IRTr* between retrieval positions in relation to a cluster switch (*Switching_IRTr*).

To explore the relationship between *IRT* and *IRS*, we ran a mixed model for the responses classified as clustering and switching. The mixed model explored whether the *IRT* values predict the *IRS* values. We included the rank of each word in the model to control for time on task, and included the subjects and cue words as random effects.

To examine how our four types of responses relate to creative abilities and executive function abilities, we ran Spearman correlations between executive function tests and creative abilities tests and *Fast-Clustering*, *Fast-Switching*, *Slow-Clustering*, and *Slow-Switching*. Spearman correlations were chosen due to the non-normal distribution of most variables, except for *CAT_CR*, *Stroop-interference*, and *Fast*-*Clustering*, which minimized the impact of outliers and data skewness. Despite these variables being normally distributed, the correlation coefficients and *p*-values yielded similar results to those obtained using Pearson correlation, in terms of statistical significance. We used false discovery rate (FDR) to correct for multiple comparisons.

#### 2.1.9 MRI data acquisition and preprocessing

Brain data were available only for the original study (Study 1). The acquisition and preprocessing of the MRI data are the same as described in Ovando-Tellez, Kenett, et al. (2022). Briefly, MRI data were collected using a 3T scanner while participants performed the relatedness judgment task. Functional and structural images were preprocessed using standard pipelines for multi-echo EPI acquisition, including denoising, motion correction, co-registration, and normalization to MNI space. Residual BOLD signals were extracted using a general linear model to remove task-related effects and motion artifacts, then concatenated across runs for further analysis. Detailed acquisition and preprocessing procedures are provided in Supplementary Methods S5.

#### 2.1.10 Predicting behavior from the individual’s brain functional connectivity patterns

##### 2.1.10.1 Building functional connectivity matrices

For each participant, we built a 200 x 200 functional connectivity matrix. To this aim, we first defined our brain regions of interest. We used the Schaefer atlas (Schaefer et al., 2018), composed of 200 brain regions distributed in 17 functional networks. Using Nilearn v0.3 (https://nilearn.github.io/) in Python 2.7, we explored the BOLD signal in these regions and correlated them across time. The 200 regions of interest were used as rows and columns to build the individual matrices, and the Pearson correlation coefficients between each pair of regions were used to fill the corresponding matching cells. Therefore, each matrix represents the individual brain network, with the nodes represented by the 200 regions of interest connected by links represented by the correlation coefficients.

##### 2.1.10.2 Connectome brain predictive modeling (CPM)

To predict individual patterns of foraging behavior, we used the CPM approach with leaving-one-out cross-validation (Shen et al., 2017). We ran four independent CPM analyses to predict *Fast-Clustering*, *Fast-Switching*, *Slow-Clustering*, and *Slow-Switching*, respectively. The CPM consisted of three steps. In the first step, we selected the significant features related to the foraging behavior patterns. For N-1 participants, we correlated each link of their brain network (i.e., each value in the individual’s brain connectivity matrix) with the behavior to be predicted. The links that correlated positively or negatively with the behavior (*p* < .01) were saved as positive and negative masks, respectively. In the second step, for each participant (i.e., N-1), we summed the weights of all the links that composed the positive and negative mask, which resulted in one unique value for each mask for each participant (i.e., mean connectivity strength). In the third step, we used these values to fit a linear regression model as predictors of behavior. In addition, we added a third predictor, which was the participants’ mean framewise displacement (Power et al., 2014) during the acquisition. This parameter controls the head movements inside the scanner during the data acquisition and ensures that the prediction is not biased by noise. In the last step, we tested this model in the left-out subject to predict its behavior. We ran these steps N times (N = 86). For each loop, the positive and negative masks were slightly different, so in the end, the final positive and negative masks were composed of brain links that were significantly correlated to the behavior in all the loops. We used the BrainNet toolbox implemented in MATLAB (R2017b, The MathWorks, Inc., USA) to visualize the predictive networks.

#### 2.1.11 Mediation analysis

To test whether the patterns of functional connectivity that predict individual patterns of memory search are also relevant for creativity, we ran mediation analyses. For significant CPM predictions, we tested whether the frequency of the types of responses during semantic retrieval mediates the relationship between their predictive patterns of brain functional connectivity and creativity. The mediation analyses focused on the types of responses that were predicted by the CPM and that correlated with creativity scores. In all analyses, we used the positive predictive brain network because we expected positive correlations with the creativity score. The mediation analyses explored an indirect effect of functional brain connectivity on creativity through the semantic memory search patterns. The mediation analysis (Hayes, 2018) quantified the effect of brain connectivity on the semantic memory search patterns (path a), the effect of the semantic memory search patterns on creativity controlling for brain connectivity (path b), the total effect of brain connectivity on creativity (path c), and direct effect of brain connectivity on creativity controlling for the semantic memory search patterns (path c’). The indirect effect was calculated as the product of path a and path b. All the variables entered in the mediation analyses were normalized. We tested the significance of the indirect effect using a bootstrapping method, computing unstandardized indirect effects for each 5000 bootstrapped samples, and the 95% confidence interval was computed by determining the indirect effects at the 2.5th and 97.5th percentiles. The mediation analyses were performed using MATLAB codes (R2017b, The MathWorks, Inc., USA).

### 2.2 Study 2

We conducted an independent replication study of the behavioral results in a new dataset, using a computerized version of the PolyFT with an extended verbal material. The experimental design and all variables used in this study are summarized in Figure 1 and Table 1.

#### 2.2.1 Participants

We collected data from 114 French native speakers who performed the same set of tasks as in the original Study 1, except for the digit span test. The inclusion and exclusion criteria from Study 1 were also applied to this study. Participants also performed the GrefSem French naming test (Merck et al., 2011), to ensure an intact semantic memory. Participants who scored below the pathological threshold on this test were excluded from the analysis. The final sample consisted of 92 participants (47 women, mean age = 27.78 years, SD = 5 years). Participants gave informed written consent and received monetary compensation for their participation. The study was approved by an approved ethics committee.

#### 2.2.2 General procedure

Participants completed computerized versions of the tasks at the Paris Brain Institute’s experimental platform PRISME classroom, allowing computer testing for up to 12 participants simultaneously. We used the same set of behavioral tasks as in Study 1, except for the digit-span test. The methods for the AUT and CAT in this study are the same as those described for Study 1. The only difference is that Study 2 used only a subset of 40 trials for CAT. Due to technical issues, five participants did not complete the AUT and CAT. The PolyFT and executive tests were similar but adapted for a computerized version as described below.

#### 2.2.3 Computerized version of the polysemous associative fluency task (PolyFT)

We developed a computerized version of the PolyFT. Unlike the original study, where participants responded orally, in this version, cue words were displayed in the center of the screen, and participants typed their responses using the keyboard. To account for typing time, we extended the duration of trials from 1 minute (as in Study 1) to 1.5 minutes. Participants were presented with the 3 cue words from the original study, plus 47 new ones, providing a total of 50 PolyFT trials in Study 2. The new cues were selected following the same criteria as described in Study 1. We selected words with at least three different meanings according to the French linguistic resource for research (Centre National de Ressources Textuelles et Lexicales; https://www.cnrtl.fr/). The 50 cue words of the PolyFT were distributed across 4 blocks (12 trials for 2 blocks and 13 trials for 2 other blocks), with equivalent average lexical frequency and number of different meanings across cues between blocks. After data collection, we excluded one cue word from the analysis since more than 25% of participants did not switch for this cue. Thus, the final number of PolyFT trials that were analyzed is 49. Participants’ responses were corrected for spelling, homogenized in terms of gender and singular form, and manually categorized within one meaning of the cue to identify switching responses. As in Study 1, we computed each participant’s *PolyFT_fluency* as the mean number of unique responses produced for each cue.

#### 2.2.4 The inter-response time and similarity between retrieved words

We computed the *IRT*s as the delay between the validation of the previous response (keypress of the “enter” key or beginning of the trial for first response) and the keypress of the first letter of the current response. To adjust the *IRT*s for the time participants spent thinking while writing, we computed the typing speed for responses longer than four characters. For each participant, we used their fastest typing speed to estimate the theoretical typing duration for each response. The difference between the measured and theoretical typing durations was considered as thinking time and was added to the *IRT* value. We computed the inter-response semantic similarity using the Word2Vec model from Polytechnique (http://nlp.polytechnique.fr/word2vec) (Abdine et al., 2021), following the same method as described in the original Study 1.

#### 2.2.5 Computerized executive function test

Similar to the original study, we used the trail-making test to assess participants’ set-shifting abilities (Reitan, 1992). The task is the same as the paper version described previously, except that numbers and letters were displayed on the screen, and participants used the computer mouse to link the items. Participants received training and feedback for each item selected, with the background of the item turning gray for correct sequences. Additionally, the previous sequence needed to be correct to complete the next sequence. The participants had a time limit of 5 minutes to complete the task.

The participants also performed a computerized Stroop test, but due to technical issues, the data could not be analyzed.

#### 2.2.6 Statistical analysis

We performed the same analyses as in Study 1 to explore optimal foraging in semantic memory, examining *IRT* and *IRS* within and between clusters, and identifying individual patterns of fast and slow clustering and switching responses. To explore the relationship between memory search components, executive function abilities, and creative abilities, we conducted one-tailed Spearman correlations between the variables that were significantly correlated before FDR correction in Study 1. We aimed to assess the reliability of the initially observed correlations and to replicate those findings.

## 3 Results

### 3.1 Testing the marginal value theorem: consistency of semantic retrieval patterns during the polysemous fluency task—PolyFT

#### 3.1.1 The marginal value differentiates clustering and switching responses

**Fig. 2.**
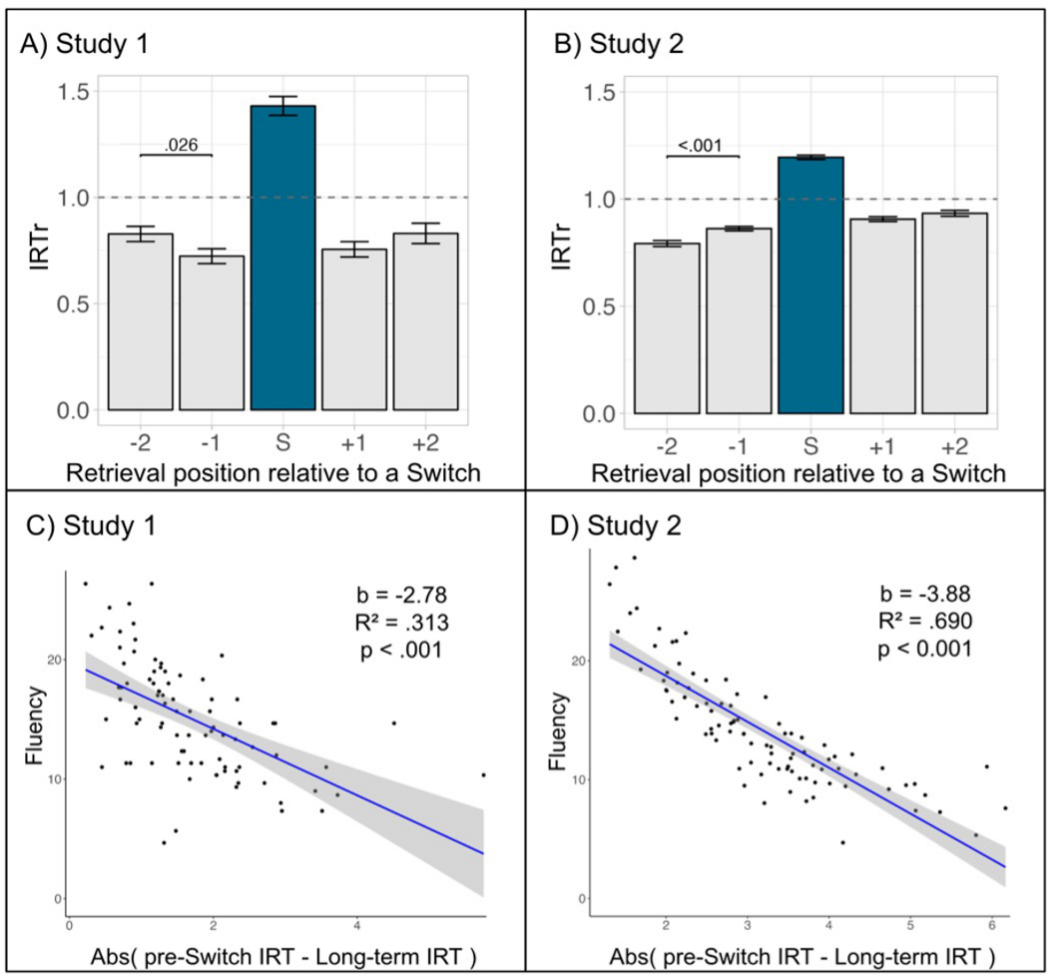
Optimal semantic foraging during PolyFT. (A, B) Values for *IRTr* are shown for each retrieval position in relation to switching (S) for Studies 1 (A) and 2 (B). The blue bar represents the values for the switching response. The horizontal dashed line represents the *long-term IRT*. Error bars indicate the standard error of the mean. (C, D) Scatter plots showing the regression of the absolute difference between the *IRT* of the responses in the -1 position (i.e., *pre-switch IRT*) and the *long-term IRT* (x-axis) on the total number of responses retrieved for the whole PolyFT (y-axis) for Studies 1 (C) and 2 (D).

During the PolyFT, participants were presented with three consecutive polysemous cue words and instructed to freely generate single-word associations related to each cue word for 1 minute per cue. Based on the meanings of the cue words (see Supplementary Table S1), we classified responses as clustering when they referred to the same meaning of the cue as the previous response, and as switching when they referred to a different meaning. To determine whether memory search patterns during the PolyFT are consistent with the MVT for semantic memory (Hills et al., 2012), we first explored whether the marginal value separates clustering and switching responses. We computed the marginal value as the ratio between the *IRT* for retrieving a word and the *long-term IRT* (*IRTr*). Consistent with this theory, in Study 1, *Switching_IRTr* was significantly greater than *Clustering_IRTr* (*W* = 3558, *p* < .001). In addition, when analyzing the *Clustering_IRTr* values for each position relative to a switching response, the mean *IRTr* for clustering responses before and after switching responses was lower than 1, while it was greater than 1 for switching responses (with 1 representing the *long-term IRT* value) (Fig. 2A). The difference between *Switching_IRTr* and *Clustering_IRTr* was significant across all clustering response positions (*p* < .001).

These results were replicated in Study 2 with an independent sample, where participants performed the PolyFT with 49 cue words instead of 3 in Study 1 (see Supplementary Table S2). In Study 2, *Switching_IRTr* was significantly greater than *Clustering_IRTr* for all clustering responses, regardless of their position (*W* = 4277, *p* < .001). The mean *IRTr* was greater than 1 for switching responses, while it was lower than 1 for clustering responses (Fig. 2B). The difference between *Switching_IRTr* and *Clustering_IRTr* was significant across all clustering response positions (*p* < .001). All descriptive statistics for Study 1 and Study 2 are available in Table 3 and Supplementary Table S3, and results from statistical tests are provided in Supplementary Table S4.

**Table 3.** Descriptive statistics for the PolyFT.

	Study 1		Study 2	
	Mean	S.D.	Mean	S.D.
*PolyFT fluency*	15.1	4.7	14.2	4.9
*Long-term IRT*	3.57	1.40	5.47	1.9
*Long-term IRS*	0.19	0.05	0.16	0.02
*Switching_IRT*	4.87	1.97	6.40	2.05
*Clustering_IRT*	2.89	1.49	4.64	1.56
*Switching_IRS*	0.06	0.05	0.09	0.02
*Clustering_IRS*	0.25	0.06	0.1	0.02

The table reports the mean and standard deviation (S.D.) for the number of responses (*PolyFT fluency*)*,* inter-response times (*long-term IRT*), and inter-response semantic distances (*long-term IRS*), computed across all responses, as well as separately for clustering and switching responses. Values were first averaged at the response level, then across the cue words and participants.

To better characterize clustering and switching responses, we analyzed *IRT*s and *IRS*s between successive responses (see Section 2.1.4.2) within and between clusters. Study 1 and Study 2 revealed longer *IRT*s between clusters than within-cluster transitions (see Supplementary Figures S1–S2 and Supplementary Results S1), as expected. We also confirmed that responses within clusters were more semantically similar than those between clusters ( Supplementary Results S2). Opposite to the pattern observed for *IRTr,* participants reached their average semantic retrieval (mean *IRS* in the entire task) during clustering responses, whereas in switching responses, they retrieved more dissimilar or distant concepts than the average (see Supplementary Figure S3 and Supplementary Results S3). These results indicate that memory search patterns during the PolyFT are consistent with the predictions of the MVT for semantic memory.

Furthermore, we analyzed the dynamics of *IRTr* before switching responses, predicting that *Clustering_IRTr* at position -1 (*pre-switch IRT*) would be significantly closer to the *long-term IRT* than at position -2. As expected, and predicted by the MVT, the last clustering response before a switch has a higher *Clustering_IRTr* than the previous one, suggesting an *IRT*s increase as participants approach a switch ( Supplementary Results S4).

#### 3.1.2 Efficiency in memory retrieval: leaving a cluster when reaching the marginal value

Following Hills et al. (2012), we explored whether participants with *IRT* close to reaching their *long-term IRT* before switching generated more responses during the task (higher *PolyFT_fluency*), as would be expected from the MVT. The value of the *Clustering_IRTr* before switching (responses in position -1 in Fig. 2A, B) reflects how close the participants are to their *long-term IRT.* We regressed the absolute difference between the *IRT* in position -1 and the *long-term IRT* on the total number of responses retrieved (averaged across the PolyFT cue words for each participant). The results from Study 1 show that the closer participants were to their *long-term IRT* before switching, the higher the *PolyFT_fluency* (*R*^2^ = 0.313, *F*(1,84) = 39.66, *p* < .001; Fig. 2C).

This finding was replicated in Study 2 (*R*^2^ = 0.690, *F*(1,90) = 214.3, *p* < .001; Fig. 2D). Similar results were observed in a control analysis using the uncorrected *IRT*s (see Supplementary Figure S4 and Supplementary Results S5), supporting the expectations from the MVT that participants with *IRT* close to reaching their *long-term IRT* before switching produce more responses during the PolyFT.

#### 3.1.3 Responses with longer IRT are more semantically dissimilar

**Fig. 3.**
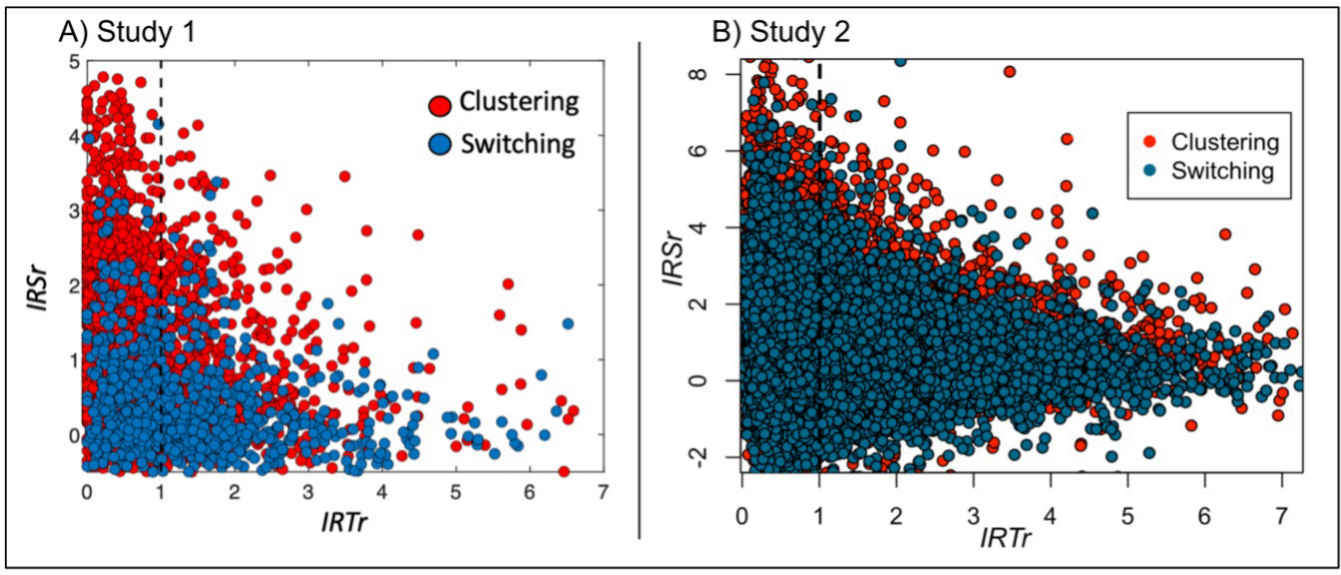
Relationship between time and similarity during semantic retrieval. We plot the relationship between time (*IRTr*) and semantic similarity (*IRSr*) for Study 1 (A) and Study 2 (B) for clustering responses in red and switching responses in blue. The vertical dashed line represents the individual *long-term IRT*. Items retrieved faster than the *long-term IRT* appear on the left of this line (*IRTr* > 1); those produced slower are on the right. Note that, for visualization purposes, plots use *IRTr* and *IRSr* instead of *IRT* and *IRS* as used in the analyses.

We further examined the link between *IRS* and *IRT* for clustering and switching responses. Mixed regression models in Study 1 showed that time and similarity correlated significantly for clustering and switching responses (clustering: *R*^2^ = 0.08, *b* = -.018, *p* < .001; switching: *R*^2^ = 0.07, *b* = -.004, *p* < .01; Fig. 3A).

We replicated these results in Study 2 (clustering: *R*^2^ = 0.06, *b* = -.008, *p* < .001; switching: *R*^2^ = 0.05, *b* = -.003, *p* < .001; Fig. 3B). Together, these results indicate that it takes longer to retrieve more distant responses both within and between clusters.

### 3.2 Switching slow but also fast: Not all responses follow the MVT

While *IRT* and *IRS* supported MVT on average (see Table 1 for a summary), participants varied in their adherence to the MVT policies. As shown in Figure 3, some switching responses occurred faster than expected by the MVT (*IRTr* < 1), whereas some clustering responses occurred later than expected (*IRTr* > 1). Therefore, we further subdivided our clustering and switching responses to account for this incongruency. We used the *IRTr* of each clustering and switching response to classify them either as fast switching or fast clustering responses (when *IRTr* was lower than 1), or as slow switching or slow clustering responses (*IRTr* larger than 1) (Table 4).

**Table 4.** Descriptive statistics of the different types of responses.

	Study 1			
	Number of responses	% of responses	*IRT* (sec)	*IRTr*
*Fast-Clustering*	7.04 ± 3.18	49.98 ± 9.95	0.82 ± 0.17	0.40 ± 0.11
*Fast-Switching*	1.73 ± 0.75	14.43 ± 7.82	2.90 ± 1.49	0.53 ± 0.14
*Slow-Clustering*	2.85 ± 1.59	20.17 ± 7.17	6.50 ± 2.92	1.96 ± 0.44
*Slow-Switching*	1.95 ± 0.78	15.42 ± 6.95	7.79 ± 3.98	2.22 ± 0.55

	Study 2			
	Number of responses	% of responses	*IRT* (sec)	*IRTr*
*Fast-Clustering*	5.65 ± 2.50	40.2 ± 5.7	2.44 ± 0.78	0.47 ± 0.04
*Fast-Switching*	2.92 ± 0.98	23.7 ± 5.4	2.83 ± 1.02	0.53 ± 0.04
*Slow-Clustering*	2.56 ± 1.21	17.9 ± 3.2	9.82 ± 3.19	1.88 ± 0.17
*Slow-Switching*	2.33 ± 0.88	18.2 ± 3.6	11.11 ± 3.56	2.07 ± 0.21

The table presents the mean ± S.D. for number of responses, percentage relative to the total number of responses, *IRT* (sec), and *IRTr* for *Fast-Clustering*, *Fast-Switching*, *Slow-Clustering*, and *Slow-Switching*. Values are provided separately for Study 1 and Study 2.

**Fig. 4.**
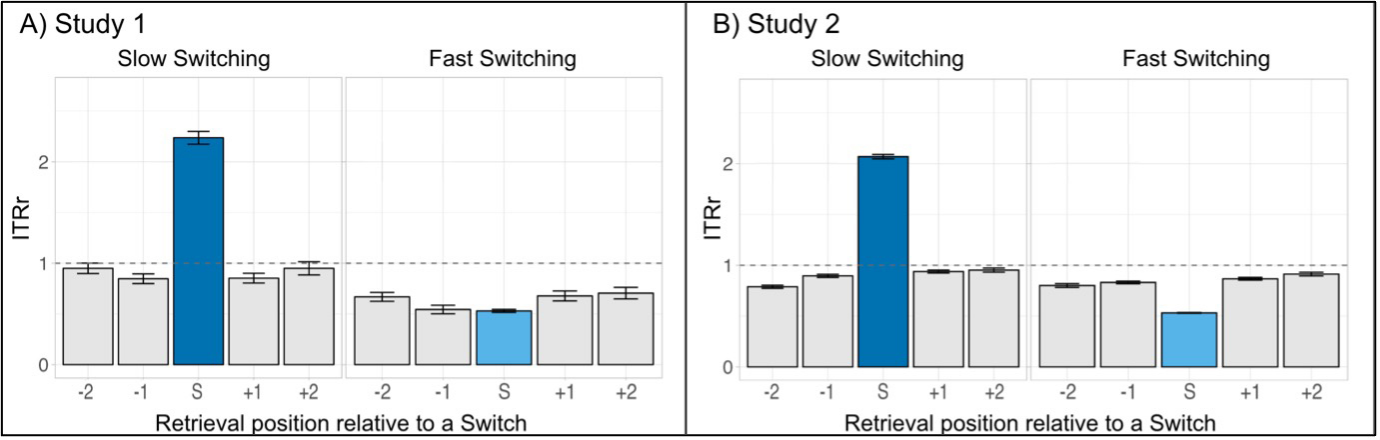
Patterns of *IRTr* are separated for slow switching and fast switching responses during semantic retrieval. Values for *IRTr* are shown for each retrieval position in relation to a cluster switch for slow switching and fast switching responses for Study 1 (A) and Study 2 (B). The dark blue and light blue bars represent the values for slow switching and fast switching responses, respectively. Position -1 represents the last clustering response before a switch. The horizontal dashed line represents the *long-term IRT*. Error bars indicate the standard error of the mean.

We then, verified that slow switching responses follow the predictions of the MVT, while fast switching responses do not. We ran the same analyses as described above (see Fig. 2), separating fast switching and slow switching responses (Fig. 4). As for the global switching responses, slow switching responses followed the expectation of the MVT in Study 1 and Study 2.

In Study 1, slow switching responses had a mean *IRTr* greater than 1, while it was lower than 1 for clustering responses before and after the switch (Fig. 4). When considering only slow switching responses, the *Clustering_IRTr* in all positions was significantly inferior to *Switching_IRTr* (*p* < .001; see Supplementary Table S5 and Supplementary Results S4). Similarly, in Study 2, the mean *IRTr* for slow switching responses was greater than 1, while it was lower than 1 for clustering responses before and after the switch (Fig. 4). We also observed that *Clustering_IRTr* in all positions was significantly lower than *Switching_IRTr* (*p* < .001; see Supplementary Table S5 and Supplementary Results S4).

When considering only fast switching responses, we observed a different pattern. In both studies, the mean *IRTr* for fast switching responses was lower than 1, as was the clustering responses before and after the switch (Fig. 4). In Study 2, we also observed that *Clustering_IRTr* in all positions was significantly higher than *Switching_IRTr* (*p* < .001; see Supplementary Table S5 and Supplementary Results S4)*.* All descriptive statistics for Study 1 and Study 2 are provided in Supplementary Table S3.

Finally, we analyzed the dynamics of *IRTr* before slow and fast switching responses, predicting that *Clustering_IRTr* at position -1 (pre-switch) would be significantly closer to the *long-term IRT* than at position -2 only for slow switching responses. As expected, and predicted by the MVT, *Clustering_IRTr* increases before slow switching responses, suggesting a ramping effect where the *IRT*s increase as participants approach a switch. This pattern was not observed for fast switching responses, where *Clustering IRTr* either decreased or remained stable before switching (Fig. 4; Supplementary Results S4).

Altogether, these findings suggest that fast and slow switching rely on distinct search mechanisms.

### 3.3 Relationships between fast and slow switching and executive control tests

To examine how fast switching and slow switching involve distinct processes (Table 1, hypothesis 2), we ran Spearman correlations between executive function abilities and *Fast-Switching* and *Slow-Switching*. In Study 1, we found significant correlations between *Slow-Switching* and better abilities in executive function tasks measuring working memory (*Backward-*span: *r*_s_ = .261, *p* = .015), task shifting (*TMT-shifting*: *r*_s_ = -.232, *p* = .032), and inhibition (*Stroop-interference: r*_s_ = -.257, *p* = .018). No significant correlations were found between executive function abilities and *Fast-Switching*.

Although correlation analyses in Study 1 were not corrected for multiple comparisons, Study 2 partly replicated them, revealing significant correlations of *Slow-Switching*, but not *Fast-Switching*, with participants’ shifting abilities assessed on the Trail Making Test (*TMT-Shifting*: *r*_s_ = .23, *p* < .001), which remained significant after correction for multiple comparisons. These findings suggest that slow switching relates to shifting abilities.

Contrary to our expectations, we did not observe significant correlations between *Slow-Clustering* and executive control tests (see Supplementary Results S6). Descriptive statistics and Spearman correlation coefficients with executive function abilities are provided in Supplementary Tables S6 and S7.

### 3.4 Individual patterns of brain functional connectivity predict fast switching and slow clustering

To explore the brain correlates of our memory search patterns, we performed four independent connectome predictive modeling (CPM) analyses, using the participants of Study 1 who underwent an MRI. These analyses aimed to determine whether individual brain functional connectivity patterns could predict *Fast-Clustering*, *Fast-Switching*, *Slow-Clustering*, and *Slow-Switching*. For each participant, we built individual 200 x 200 functional connectivity matrices using the Schaefer atlas (Schaefer et al., 2018), which captures BOLD signal correlations across predefined brain regions of interest. We identified significant connections related to each response type using cross-validated linear models, generating positive and negative predictive networks. The CPM analyses revealed that brain functional connectivity significantly predicted *Fast-Clustering* (*r*_s_ = .23, *p* = .036), *Fast-Switching* (*r*_s_ = .33, *p* = .002), and *Slow-Clustering* (*r*_s_ = .33, *p* = .002) but not *Slow-Switching* (*r*_s_ = -.18, *p* = .09). After permutation testing, only *Fast-Switching* (*p* = .019) and *Slow-Clustering* (*p* = .024) remained significant. We then characterized the predictive networks for these two response types.

### 3.5 Characterization of the brain networks predicting fast switching and slow clustering

**Fig. 5.**
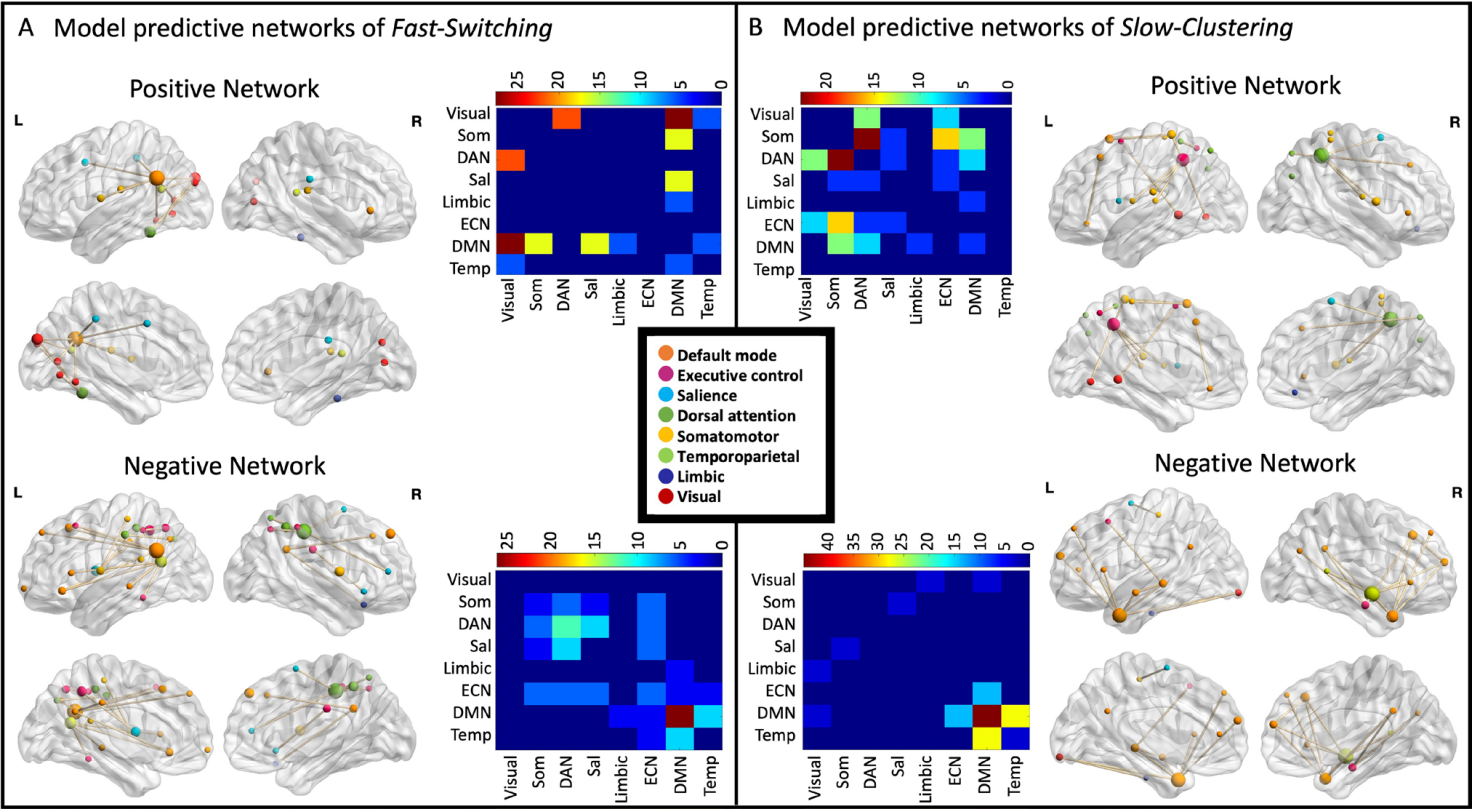
Brain networks predicting *Fast-Switching* and *Slow-Clustering*. Positive and negative predictive networks are shown for *Fast-Switching* (A) and *Slow-Clustering* (B). The nodes of the networks are color coded by the functional network they belong to. The matrices represent the percentage of edges connecting nodes of the different functional networks. L = left; R = right; Som = somatomotor network; DAN = dorsal attention network; Sal = salience network; ECN = executive control network; DMN = default mode network; Temp = temporoparietal network.

The positive model network predicting *Fast-Switching* was composed of 19 brain regions connected by 18 links (Fig. 5A). Most of these connections were localized between brain regions of the default mode and visual networks (5 links), dorsal attention and visual networks (4 links), default mode and somatomotor networks (3 links), and default mode and salience networks (3 links). The highest degree node was localized in the left inferior parietal lobule (*k* = 12), region that belong to the default mode network. This brain region connects to brain regions of the visual, somatomotor, salience, limbic, and temporoparietal networks.

The model network predicting lower *Fast-Switching* was composed of 37 brain regions connected by 34 links. Most of these connections were localized between brain regions within the default mode network (9 links), within the dorsal attention networks (4 links), between the dorsal attention and salience networks (3 links), and between default mode and temporoparietal networks (3 links). The highest degree nodes were localized in the left inferior parietal lobule (*k* = 6), region that belong to the default mode network, and the right post central region of the dorsal attention network (*k* = 6). The left inferior parietal lobule had connections to brain regions of the default mode network, and one connection to the lateral prefrontal cortex of the executive control network. The right post central region connected to regions of the dorsal attention, executive control, and salience networks.

The positive network model predicting *Slow-Clustering* was composed of 28 brain regions connected by 26 links (Fig. 5B). Most of these connections were localized between brain regions of the dorsal attention and somatomotor networks (6 links), the executive control network and somatomotor networks (4 links), the dorsal attention and visual networks (3 links), and the default mode and somatomotor networks (3 links). The highest degree nodes were localized in the right superior parietal lobule (*k* = 8), region that belong to the dorsal attention network, and the left intraparietal sulcus of the executive control network (*k* = 6). These nodes connected to brain regions of the visual, somatomotor, and salience networks.

The negative network model predicting *Slow-Clustering* was composed of 24 brain regions connected by 29 links. Most of these connections were localized between brain regions within the default mode network (13 links), between the default mode and temporoparietal (8 links), and between the executive control network and default mode networks (4 links). The highest degree nodes were localized in the right temporoparietal regions of the temporoparietal network (*k* = 9), and brain regions of the default mode network (left temporal: *k* = 8; right anterior temporal: *k* = 6; right dorsal prefrontal cortex: *k* = 4). All these regions had mostly within-network connections to the rest of the brain.

### 3.6 Relationships between PolyFT responses, brain connectivity, and creativity

Finally, we explored the relationship between PolyFT responses, brain connectivity, and creativity. Since individual patterns of brain functional connectivity predicted the frequency to which participants produce fast switching and slow clustering responses, we examined the relationship between their brain connectivity patterns and creative abilities. We assessed two types of creative abilities. First, the ability to combine remote associates was measured using the Combination of Associates Task (CAT), where participants were asked to find a fourth word that linked three seemingly unrelated words. This task evaluates both accurate (*CAT_CR*) and insight-related solutions (*CAT_eureka*). Second, divergent thinking abilities were measured with the Alternative Uses Task (AUT), which asked participants to generate as many alternative uses as possible for common objects, with scores reflecting the fluency (*AUT_fluency*) and originality (*AUT_uniqueness*) of their responses. Specifically, we explored whether the relationship between brain functional connectivity (i.e., mean connectivity strength of the positive model network) and creative abilities was mediated by the individual patterns of slow clustering and fast switching behavior during semantic retrieval (i.e., semantic foraging patterns). We conducted mediation analyses that focused on the indirect effect of brain connectivity predicting *Fast-Switching* and *Slow-Clustering* on creative abilities. In all mediation analyses, we calculated the indirect effect as the product of path a (i.e., the regression coefficient between brain functional connectivity and memory search patterns) and path b (i.e., the regression coefficient between memory search patterns and creative abilities). We tested the significance of the indirect effect using bootstrapping methods.

**Fig. 6.**
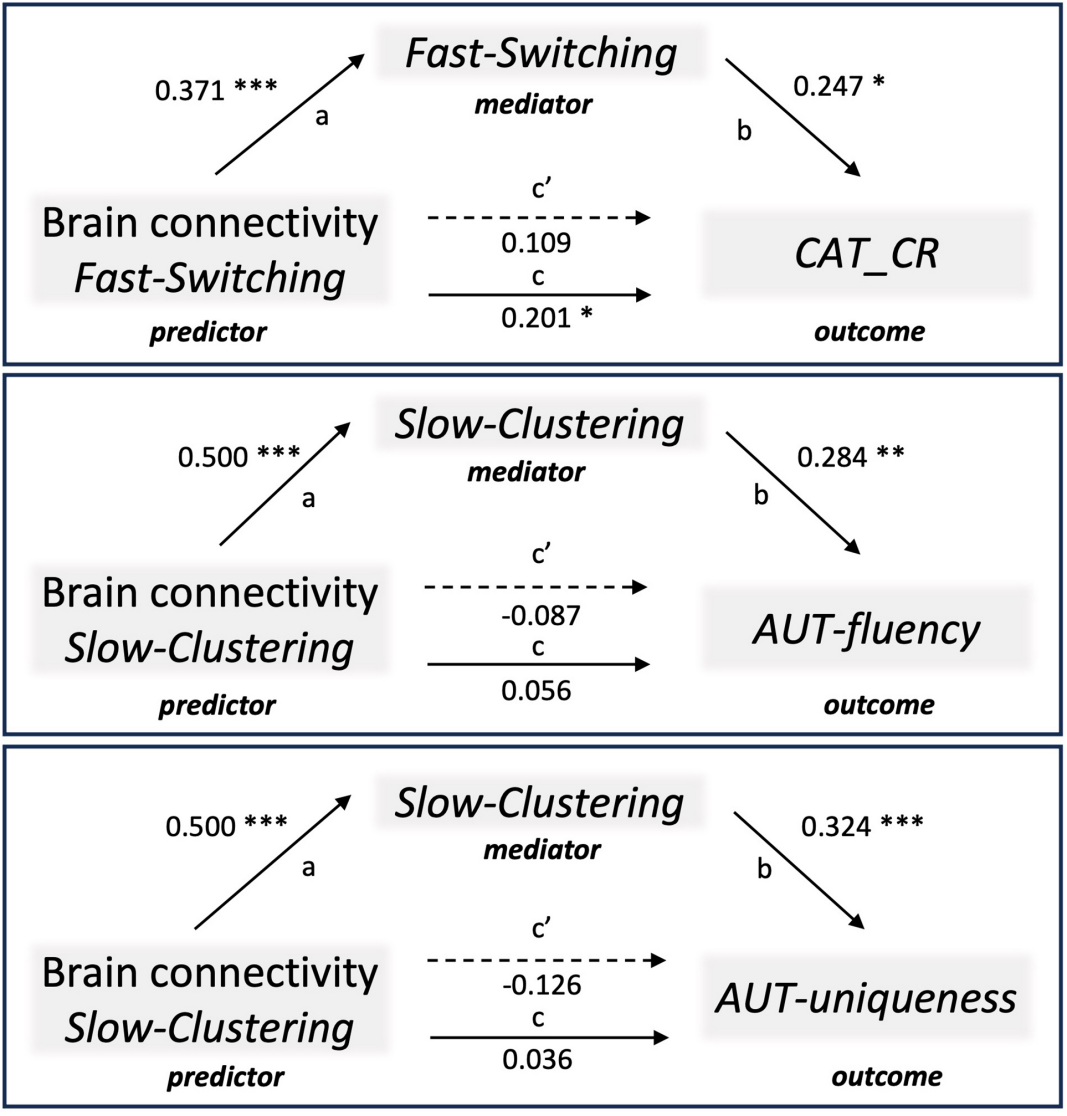
Relationship between brain connectivity, memory search types, and creative abilities. The results of the mediation models are presented in path diagrams. Each diagram indicates the beta weights of the regression coefficients, with the brain connectivity pattern of the model network (brain connectivity predictive of *Fast-Switching* and *Slow-Clustering*) as the independent variable (predictor), PolyFT responses (*Fast-Switching* and *Slow-Clustering*) as the mediator, and creative abilities to combine remote associates (*CAT_CR*) and divergent thinking (*AUT-fluency* and *AUT-uniqueness*) as the dependent variable (outcome). The total effect of the predictor on the outcome is indicated by path c, the direct effect by path c′, and the indirect effect is given by the product of path a and path b. The indirect effect was significant in all the reported mediations. *p < .05; **p < .01; ***p < .001.

Since *Fast-Switching* correlated only with the ability to combine remote associates evaluated by the CAT task in both Study 1 and Study 2, we focused our analyses on this specific association. Supplementary Table S6 reports descriptive statistics, and Supplementary Table S7 provides all correlation results between creativity measures and the occurrence of fast and slow clustering and switching. We examined the mediating role of *Fast-Switching* on the relationship between the brain connectivity patterns predicting it (predictor) and *CAT_CR* (outcome; Fig. 6, upper). As shown in the previous analyses, the regression coefficients between the brain connectivity pattern and *Fast-Switching* (*b* = .371, *p* < .001) and between *Fast-Switching* and *CAT_CR* (*b* = .247, *p* < .05) were significant. The total effect (c path) represented by the regression coefficient between the brain connectivity patterns and *CAT_CR* was significant (*b* = .201, *p* < .05), and the direct effect (c’ path) was not significant (*b* = .109, *p* = .294). The indirect effect was significant (0.371 × 0.247 = .092), with a 95% bootstrapped confidence interval [0.019, 0.206]. Hence, *Fast-Switching* mediated the relationship between the positive predictive network model of *Fast-Switching* and CAT performance: The higher the strength of connectivity in the positive network model that predicts *Fast-Switching*, the higher the number of Fast switches in semantic retrieval, and the higher the abilities to combine remote associates in memory.

We also explored the mediation analyses for *Slow-Clustering*. Because *Slow-Clustering* correlated only with the fluency and originality of the participants in the AUT in both Study 1 and Study 2 (see Supplementary Table S7 and Supplementary Results S6), we explored the mediating role of *Slow-Clustering* on the relationship between the brain connectivity patterns predicting it (predictor) and *AUT_fluency* (outcome; Fig. 6, left) and *AUT_uniqueness* (outcome; Fig. 6, right) in independent analyses. As shown in the previous analyses, the regression coefficient between the brain connectivity pattern and *Slow-Clustering* (*b* = .50, *p* < .001) was significant. The regression coefficients between *Slow-Clustering* and *AUT_fluency* (*b* = .284, *p* < .01) and between *Slow-Clustering* and *AUT_uniqueness* (*b* = .324, *p* < .001) were significant. The total effect (c path) and the direct effect (c’ path) between the brain connectivity patterns were not significant for *AUT_fluency* (total effect: *b* = .056, *p* = .549; direct effect: *b* = -.087, *p* = .390) or for *AUT_ uniqueness* (total effect: *b* = .036, *p* = .691; direct effect: *b* = -.126, *p* = .200). For *AUT_fluency*, the indirect effect was significant (0.50 × 0.284 = .142, with a 95% bootstrapped confidence interval [0.06, 0.29]), while for *AUT_originality*, the indirect effect was significant (0.50 × 0.324 = .162), with a 95% bootstrapped confidence interval [0.072, 0.32]. Hence, *Slow-Clustering* mediated the relationship between the positive predictive network model of *Slow-Clustering* and AUT performance: The higher the strength of connectivity in the positive network model that predicts *Slow-Clustering*, the higher the frequency of slow clustering responses during semantic retrieval, and the more participants were fluent and original in the divergent thinking task.

## 4 Discussion

**Fig. 7.**
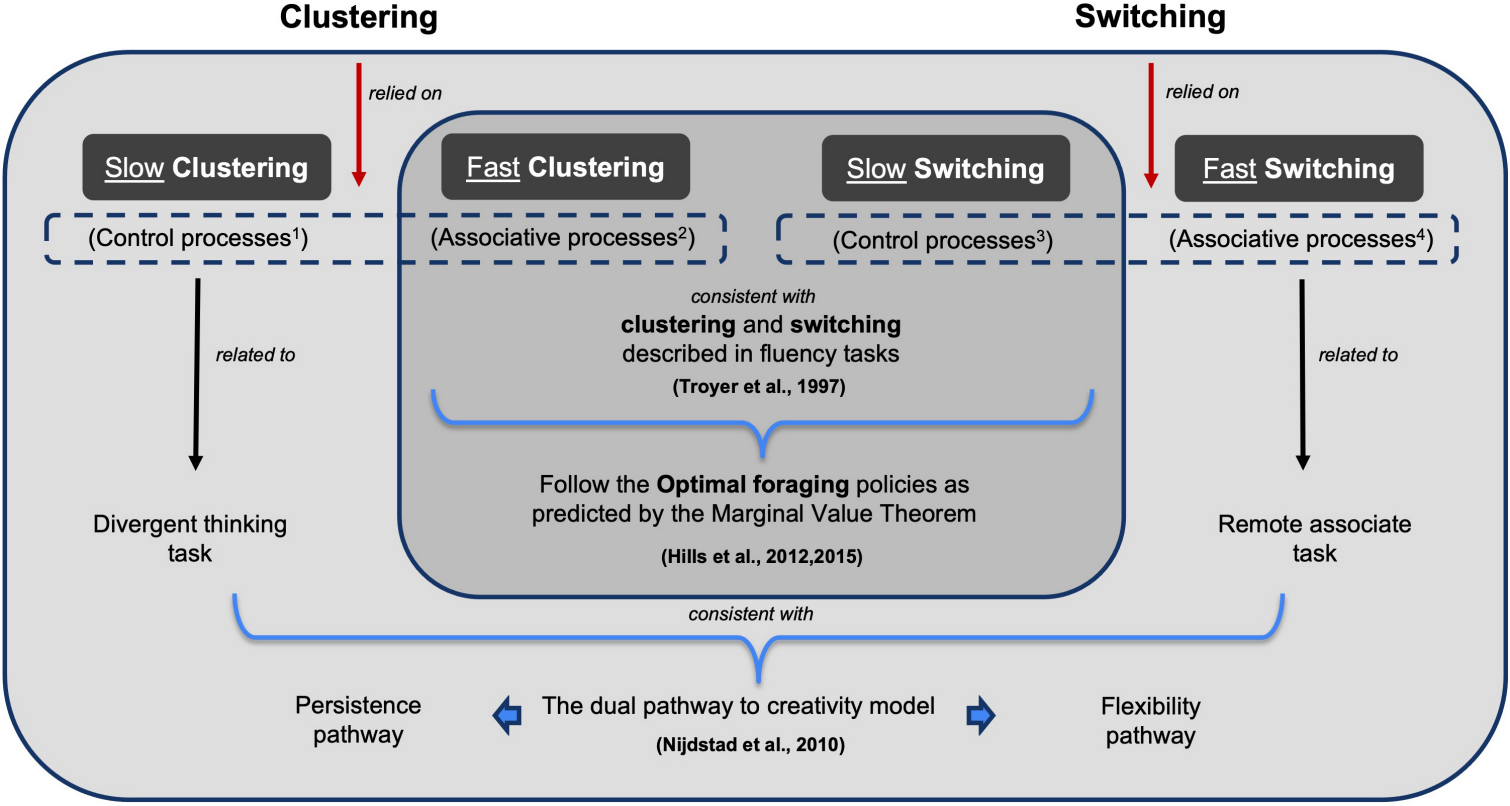
Schematic representation of results interpretations. This figure illustrates our interpretations of slow clustering, fast clustering, slow switching, and fast switching responses based on our findings at both the behavioral and brain levels. The red arrows indicate that both clustering and switching responses rely on associative and control processes. Light gray: slow clustering involves control processes and is related to divergent thinking tasks. Fast switching involves associative processes and is related to the remote associate task. These patterns are consistent with the dual pathway to creativity model, linking slow clustering and fast switching to the persistence and flexibility pathways, respectively. Dark gray: fast clustering involves associative processes, while slow switching involves control processes. These patterns align with clustering and switching described in classical fluency tasks and follow the optimal foraging policies as predicted by the MVT. Note: ^1^suggested by brain predictive patterns but not correlated with executive control tasks; ^2^hypothesized but not tested; ^3^correlation with *TMT-shifting*; ^4^suggested only by brain predictive patterns.

Processes occurring during searching in memory are critical for creativity but remain to be clarified (Kenett et al., 2023). This study characterized distinct foraging behaviors during semantic search and retrieval, explored their brain correlates, and their relationships with creative abilities and executive function abilities. Converging results from two datasets confirmed our core hypothesis: during PolyFT, individuals generally follow an optimal foraging policy consistent with the MVT. However, not all responses adhered to this policy, with some switches occurring faster and some clustering responses occurring slower than MVT predictions. The identification of such patterns provides new hypotheses and measures for investigating the processes involved in semantic foraging. The differentiation between these fast and slow responses allows for reconciling prior theories and approaches, as these response types likely reflect distinct underlying cognitive processes or strategies. Specifically, fast clustering and slow switching are consistent with MVT principles and with the classical distinction of clustering and switching in fluency tasks (Troyer et al., 1997). In contrast, fast switching and slow clustering are relevant to creativity and may align with the flexibility and persistence pathways of the “two pathways to creativity” model (Nijstad et al., 2010). We further identified functional connectivity patterns predictive of the frequency of fast switching and slow clustering responses, revealing that fast switching between clusters (which might be related to spontaneous processes) mediates the relationship between brain connectivity and the ability to combine remote associates in memory, whereas slow clustering (which might be related to controlled processes) mediates the relationship between brain connectivity and divergent thinking abilities (Table 1; Fig. 7).

Although different accounts of how people search in memory for concepts exist (Abbott et al., 2012; Todd & Hills, 2020; Troyer et al., 1997), studies using classical fluency tasks have shown that memory retrieval involves the interaction between automatic and controlled processes, possibly supporting clustering and switching, respectively (Hills & Pachur, 2012; Hills et al., 2013; Lundin et al., 2023; Rosen & Engle, 1997; Troyer et al., 1997). The MVT describes these interactions as local–global transitions related to processes of exploitation–exploration, in which global transitions between clusters occur when the retrieval rate within the current cluster falls below a threshold (Charnov, 1976; Hills et al., 2012, 2015). In our study, we combined the clustering–switching approach with the MVT during an associative fluency task, by analyzing the clustering and switching responses as a function of inter-response time. As expected, we observed a pattern of search behavior consistent with the MVT, in which switches between clusters occur when the time to retrieve a response reaches the individuals’ retrieval time in the entire task (i.e., *long-term IRT*). Furthermore, the closer participants were to their *long-term IRT* (their individual threshold) before switching, the more responses they retrieved during the task (Fig. 2; see also Supplementary Results S7 and Supplementary Table S8). These findings indicate that during memory search, patterns consistent with the MVT are related to fluency, reflecting the efficiency of retrieving many items. Additional analyses, replicated in both Study 1 and Study 2, showed that transitioning to another cluster (i.e., switching to a different meaning) took longer than staying within the same cluster, and the retrieved elements were more dissimilar to each other than within-cluster responses (Supplementary Results S1 and S2). It is important to note that the clusters were assigned by design. We expected words related to the same meaning to be closer in terms of semantic distance, and we aimed to capture this effect when comparing semantic similarity within and between clusters. The organization of words in semantic memory based on their meaning or category is a debated question, making our findings particularly noteworthy. Altogether, these findings demonstrated that semantic retrieval in the PolyFT follows the MVT, as previously demonstrated with classical fluency tasks (Hills et al., 2012, 2015; Todd & Hills, 2020). This broader applicability of the MVT principles beyond traditional category-based tasks suggests their wider and more general relevance in understanding how people access and retrieve information from their semantic memory.

Although the MVT prediction was appropriate on average, we also observed considerable variation in whether switches were made or not at any given point during the PolyFT. Specifically, we discovered that both clustering and switching can occur slow and fast (slower or faster than the MVT would predict; Fig. 3; Table 4). First, considering clustering, fast clustering responses are consistent with the MVT prediction, and also converge with the clustering component described in Troyer et al. (1997) using classical fluency tasks, which proposed that clustering involves spontaneous memory processes. Conversely, slow clustering responses deviate from the MVT predictions—participants are staying too long before switching—and the longer latency may suggest the involvement of more controlled processes, in terms of deliberate exploitation of semantic clusters rather than passive associations. However, contrary to our expectations, correlations between *Slow-Clustering* and the executive control tests were not significant ( Supplementary Results S6). This negative result might be explained by a lack of power in our studies. Alternatively, it is possible that the chosen tests do not capture the controlled processes involved in slow clustering, which we hypothesized to require sustained attentional control. Future studies using more appropriate tests targeting attentional focus may help clarify this result.

Importantly, we were able to predict *Slow-Clustering* from brain connectivity (Fig. 5). The brain network predicting more frequent slow clustering responses was related to higher divergent thinking ability (measured by the fluency and originality of AUT responses), mediated by more slow clustering responses (Fig. 6). The relationship between *Slow-Clustering* and creative ideation resonates with previous studies suggesting that longer response latency in divergent thinking tasks is related to increased originality and greater dissimilarity between consecutive responses (Acar et al., 2019; Hass, 2017; Mastria et al., 2021). These findings highlight the significance of thoroughly exploiting a topic to generate more and more creative ideas, which is in agreement with the persistence pathway of the dual pathway to creativity model (De Dreu et al., 2008; Nijstad et al., 2010). According to this model, the persistence pathway requires a sustained and focused cognitive effort to achieve creative ideas. Supporting this hypothesis, the brain network predicting more slow clustering responses was characterized by interactions between dorsal attention and executive control networks in conjunction with somatomotor networks. Highly connected nodes of this predictive network were localized in the right superior parietal lobule of the dorsal attention network and the left intraparietal sulcus of the executive control network. Previous studies have shown the role of intraparietal sulcus in control-demanding semantic tasks (Noonan et al., 2013) and multiple-demand tasks (Fedorenko et al., 2013), which is consistent with the proposed role of control-related networks in slow clustering. Altogether, these findings suggest that more persistent memory search behavior, supported by higher functional connectivity in attentional and control-related networks, allows better performance in divergent thinking tasks.

As for clustering, switching responses also occurred fast and slow. These two switching types, slow switching and fast switching, showed different cognitive patterns (Fig. 4). Slow switching aligned with the classical description of switching in Troyer et al. (1997) during semantic retrieval, and its cognitive pattern follows the policies of the MVT. We expected switching to be related to executive abilities and to reflect more controlled processes. As hypothesized, *Slow-Switching* was correlated to set-shifting (*TMT-shifting*; Supplementary Results S6), which is one of the key components of executive control (Miyake et al., 2000). However, correlations with working memory and inhibition tests were less reliable in our dataset. Given the ongoing debate regarding the fractionation of executive functions (Baddeley, 2002; Friedman & Miyake, 2017; Miyake et al., 2000), strategy differences (Unsworth, 2017), and the challenges associated with interpreting negative findings, our results do not conclusively establish that slow switching specifically relates to set shifting compared with other executive functions. However, they do demonstrate the involvement of controlled processes in this slow component, highlighting the complexity and multifaceted nature of executive control involved in semantic retrieval tasks. Nevertheless, these findings are consistent with previous work exploring clustering and switching responses during the generation of creative ideas. Compared with clustering, switching requires a larger investment of cognitive resources during creative ideation and involves longer search times (Hass, 2017; Mastria et al., 2021). Future studies using more refined measures and experimental designs that specifically target different aspects of executive functioning could help to further elucidate their role in semantic foraging behaviors.

In contrast, *Fast-Switching* was predicted by specific brain connectivity patterns and was relevant to creative abilities (Figs. 5 and 6). Individuals with more fast-switching responses had higher performance in the CAT, a creativity task requiring the combination of unrelated associates in memory (Ovando-Tellez et al., 2023). The link between switching and CAT is plausible, as more creative individuals have been shown to be more flexible and are more likely to activate multiple lexico-semantic meaning representations (Beaty & Kenett, 2023; Benedek et al., 2023; Mednick, 1962). The CAT involves activating different semantic representations of each of the three cue words to find the one that is shared across cue words, making semantic switches between different representations of the cue words essential to the performance of this task. Hence, fast switching may reflect the flexibility pathway of the dual pathway to creativity model (Nijstad et al., 2010), which proposes that creative ideas can be produced by flexible thinking, including spontaneous flexibility. Flexible and fast switches between categories or semantic representations may facilitate connecting more remote associates within memory and, thus, lead to a better performance in CAT.

If fast switching reflects a form of spontaneous flexibility that is relatively free from effortful control, its correlation with the CAT is consistent with the associative theory of creativity. Mednick (1962) hypothesized that more creative people had flatter associative hierarchies enabling them to connect distant concepts. In our data, fast switching responses were produced fast and were semantically remote from the previous item. We thus hypothesize that these fast switches reflect semantic shortcuts within an individual’s memory organization with respect to the typical memory organization, potentially indicating closer associations within their own semantic memory. In the PolyFT, individuals with more fast-switching responses may have shorter distances between concepts related to the different meanings of cue words, facilitating quick access to those concepts, or less modular semantic memory networks, allowing faster transitions between meanings (Ovando-Tellez, Benedek, et al., 2022). This idea aligns with Schilling (2005) theory that shortcuts in an individual’s network of representations occur during insight, that is, atypical or new associations in the individual memory organization, although we did not observe correlations between *Fast-Switching* and the insight phenomenon measured in the CAT (*CAT_eureka*). Overall, more frequent fast switching may suggest a more flexible exploration in memory that facilitates associative combination abilities.

The brain network predictive of *Fast-Switching* was dominated by default mode network connections and was characterized by an inferior parietal lobule region of the default mode network overlapping the left angular gyrus. This region was highly connected to brain regions across all other functional networks, with no links to regions within the default mode network and the executive control network. Interestingly, the brain network predicting a lower behavior of fast switching was characterized by the same node connecting with other regions within the default mode network or with the executive control network. Previous research indicates that the angular gyrus is a critical brain region for semantic cognition, as it is considered a hub region for semantic integration (Binder et al., 2009; Ralph et al., 2017) and it has been shown to be critical for the automatic retrieval of concepts in memory (Davey et al., 2015; Humphreys & Lambon Ralph, 2015; Jackson, 2021; Jefferies et al., 2020). The angular gyrus has also been associated with different creativity tasks and domains across studies (Gonen-Yaacovi et al., 2013). Moreover, the literature has widely supported the role of the default mode network regions in the spontaneous generation of associates (Davey et al., 2016; Teige et al., 2019), in judging remote concepts as related (Herault et al., 2024), and in creative abilities (Beaty et al., 2015; Bendetowicz et al., 2018; Marron et al., 2018). In sum, both behavioral and neural findings suggest that fast switching may relate to associative processes. However, whether fast switching represents purely automatic processes remains to be directly tested.

Overall, the current study clarifies and extends previous findings linking classical clustering and switching to semantic memory structure, executive control, and creative abilities (Ovando-Tellez, Benedek, et al., 2022). By analyzing the data using a different approach, in light of the MVT, we were able to advance our understanding of memory search processes in creative abilities. Here, we identify two distinct search patterns. One pattern, characterized by fast clustering and slow switching responses, is consistent with the MVT policies and with the classical interpretations of the processes underlying clustering and switching responses during searching in memory (Troyer et al., 1997). Fast clustering likely involves associative processes, while slow switching relates to controlled processes. Consistent with our first and second hypotheses, these MVT-consistent patterns were associated with greater fluency, suggesting they reflect efficient retrieval processes during the PolyFT (Fig. 2; Supplementary Results S7).

However, we introduce a second pattern including slow clustering and fast switching responses that is not following the MVT policy but relates to creative abilities and mediates the relationship between brain connectivity and creative abilities, confirming our third and fourth hypotheses. Theoretically, slow clustering and fast switching responses deviate from MVT policy but may align with the dual pathway to creativity model (Nijstad et al., 2010, also see Benedek, 2024), which proposes persistence and flexibility as distinct paths for creative idea generation. In this model, persistence refers to an effortful, sustained search involving cognitive control, while flexibility refers to generating ideas by shifting between categories or perspectives, potentially involving automatic or controlled processes, depending on task demands and individual differences. We propose that slow clustering captures a persistent local semantic search to maximally exploit a cluster, requiring cognitive control, reflected in the involvement of the executive control and attentional networks, and is critical for divergent thinking. Conversely, fast switching reflects an associative form of flexible search across various semantic clusters, which seems to involve the default mode network, and relates to the ability to combine remote concepts (as measured by the CAT). These findings are consistent with recent evidence showing that retrieval flexibility mediates the link between brain dynamics and creativity (Zhang et al., 2023). Taken together, these findings refine existing models by suggesting that creativity can emerge not only from the interaction between default mode network and frontoparietal control network (Beaty et al., 2015) but also from the flexible recruitment of distinct network configurations depending on the type of cognitive search strategy.

Importantly, while MVT-consistent patterns (i.e., fast clustering and slow switching) maximize fluency in the PolyFT, MVT-deviant patterns (i.e., slow clustering and fast switching) may reflect persistence and flexibility during search that facilitate creativity, as we showed a link between these MVT-deviant patterns and greater originality of the responses in the PolyFT ( Supplementary Results S7). Notably, participants were not instructed to be creative; yet our mediation and correlation findings showed that those with more fast switching or slow clustering responses had higher creative performance on independent creativity tasks (i.e., CAT and AUT). These results support the idea that cognitive policies fostering creativity may diverge from those optimizing fluency and highlight the importance of both persistence and flexibility in creative abilities.

These findings suggest that distinct search patterns captured by the PolyFT can reveal behaviors associated with creativity, even in the absence of explicit creative instructions. In light of recent theories, individual differences in MVT-deviant patterns of search behavior may reflect stable traits, as persistence and flexibility have shown trait-like consistency across tasks (Koutstaal, 2025). Alternatively, they may emerge from biases in the recruitment of “fast” (intuitive, flexible) versus “slow” (controlled, deliberative, persistent) modes during memory search (Khalil & Brüne, 2025). Hence, memory search patterns in the PolyFT may reflect the dynamic interplay between fast (associative/ flexible) and slow (controlled/persistent) processes, shaped by both trait-like consistencies and individual biases toward persistence or flexibility. From this perspective, MVT-deviant patterns may not be considered failures of optimality in all contexts but rather alternative search dynamics that characterize more creative individuals. Identifying the policies that drive creativity, rather than fluency, during memory search is an avenue for future research and implications that our results open.

Considering the established role of both associative and control processes in creativity (Beaty et al., 2014; Benedek & Jauk, 2018; Volle, 2018), one could also argue for the relevance of slow switching as well as fast clustering MVT-consistent responses in creative performance, corresponding, respectively, to the executive control and semantic memory representation involved in fluency tasks (Troyer et al., 1997). Although we did not run mediation analyses using *Fast-Clustering* and *Slow-Switching* due to their non-significant CPM models, correlations indicate a link between these behaviors and creative abilities (see Supplementary Results S6). In particular, *Fast-Clustering* correlated significantly with divergent thinking abilities. Fast clustering may reflect spontaneous associative retrieval in memory, consistent with Troyer’s hypothesis, and their relationship with creative abilities is consistent with the role of memory structure in creativity (Beaty & Kenett, 2023; Benedek et al., 2023; Kenett & Faust, 2019; Mednick, 1962). Although their correlations with *Slow-Switching* were less consistent, the current findings do not exclude the possibility that MVT-consistent patterns of fast clustering and slow switching also play a role in creative thinking. In fact, additional analyses on the PolyFT testing the role of MVT-consistent and MVT-deviant patterns in creative abilities suggest that slow clustering and fast switching may be indicators of creativity beyond MVT-consistent patterns (see Supplementary Results S7).

We acknowledge some limitations of our study. First, our results were obtained using polysemous words in the French language, which may limit the generalizability of our findings. The use of polysemous cues introduces semantic competition between alternative meanings, but it also offers a methodological advantage as it allows clearer identification of clustering and switching dynamics. The similarity of our clustering and switching responses to those observed in classical fluency (Troyer et al., 1997, 1998) and creativity tasks (Acar & Runco, 2017; Hass, 2017; Mastria et al., 2021; Nusbaum & Silvia, 2011) suggests that the underlying search mechanisms may be at least partly generalizable (see also Hills et al., 2015; Koutstaal, 2025; Mekern et al., 2019). Moreover, polysemous words may be particularly suited for studying creativity, as they require navigating between multiple meanings, a process closely related to representational flexibility and tolerance for ambiguity, both of which have been linked to creative cognition (Atchley et al., 1999; Best et al., 2015; Blake & Palmisano 2021; Wiseman et al., 2011). Nevertheless, the use of the French language may introduce language-specific effects. Although cross-linguistic studies using polysemous words suggest a shared semantic structure across languages (Liang et al., 2024; Youn et al., 2016), language-specific semantic organization and cue ambiguity may still modulate cluster boundaries, switching thresholds, or preferred meaning sequences. Future research is required to determine whether our results can be generalized to other tasks and languages, and to determine the extent to which task instructions or language-specific factors have an impact on search dynamics.

Second, we did not account for the possible variations in *IRT*s along the task. We used a *long-term IRT* specific to each cue word to define a marginal value for each participant but did not consider its potential dynamic changes over time. It might be important to consider more dynamic measures during the PolyFT in future studies, as participants may adapt their strategies or behavior as the task progresses.

Third, we were unable to predict *Fast-Clustering* and *Slow-Switching* from brain connectivity patterns, which prevented us from comparing their brain patterns of functional connectivity with those observed in slow clustering and fast switching. A recent fMRI study by Lundin et al. (2023) revealed distinct neural patterns for clustering and switching during memory search. Their findings revealed increased activity in brain regions, such as the hippocampus and cerebellum, during exploration (as opposed to exploitation of clusters) and leading up to switches, suggesting a potential cognitive monitoring process. However, their study did not distinguish between fast and slow clustering and switching, which constitutes an avenue for future fMRI studies to explore these distinctions.

Fourth, our interpretations of slow clustering and fast switching as potentially reflecting over-exploitation and over-exploration of semantic clusters, respectively, remain speculative. We did not examine the decision-making processes underlying search transitions. Research on decision making offers useful tasks and models for this purpose. For example, the multi-armed bandit task (Cohen et al., 2007; Robbins, 1952; Wilson et al., 2014) is widely used to investigate how agents balance exploiting a current option versus exploring alternatives, while reinforcement-learning models (Kaelbling et al., 1996; Sutton & Barto, 1998) formalize the computational principles of such behavior. Applying these frameworks to semantic foraging could help interpret fast switching as a form of flexibility-driven exploration, and slow clustering as a persistent exploitation policy, both of which may reflect distinct strategies within memory search. In this context, recent work shows that the subjective value of retrieved ideas guides their evaluation and selection during creative thinking (Battistello et al., 2025; López-Persem et al., 2024; Moreno-Rodriguez et al., 2025). Search transitions in the PolyFT may, therefore, also reflect value-based decisions about when to persist or switch. Our behavioral distinction between fast and slow clustering/switching may thus be analyzed in the light of recent theoretical revisions of dual-process and decision-making theories (e.g., De Neys, 2023; Khalil & Brüne, 2025). Future work could benefit from integrating computational models of value-based decision making to further clarify the dynamics of switching behavior in relation to creativity.

Finally, the higher or lower occurrences of fast or slow clustering and switching responses could also be impacted by higher-order regulation processes. Individual’s differences may reflect flexible metacontrol-based adjustments of cognitive search (Mekern et al., 2019; Zhang, Sjoerds, & Hommel, 2020), which bias individuals toward persistence or flexibility. Such metacontrol biases have been proposed to modulate creative cognition by influencing whether individuals favor divergent or convergent thinking modes (Zhang, Sjoerds, & Hommel, 2020). Hence, the ability to switch fast or slow may be related to metacognitive monitoring and control processes, which reflect how effectively people evaluate and regulate their performance during memory search (Lebuda & Benedek, 2023; Lopez-Persem et al., 2023), impacting their creativity (Benedek & Lebuda, 2024; Lebuda & Benedek, 2024). Future research is needed to understand the metacognitive processes at play during semantic foraging in creative thinking.

## 5 Conclusions

Our findings demonstrate that the processes occurring during memory search differ between individuals and relate to creativity. By characterizing fast and slow clustering and switching, we captured more nuanced aspects of semantic foraging behavior during the PolyFT, separating behavioral responses that reflect optimal foraging from those that represent creativity‐relevant deviations. Patterns consistent with foraging principles (fast clustering and slow switching) supported efficient retrieval and fluency, while deviations from this policy (slow clustering and fast switching) mediated the link between brain connectivity and creative performance. Slow clustering is interpreted as a persistent search supported by attentional and control networks and related to divergent thinking, whereas fast switching reflected a flexible search supported by default mode connectivity and related to combining remote associations. Altogether, these findings help clarify the discrepancies between previous approaches and theories on memory search by extending classical foraging models, showing that deviations from purely optimal retrieval can be helpful for creativity and providing a framework for linking semantic search dynamics, creative thinking, and brain network organization. Moreover, our findings introduce new measures of memory search behavior that can help advance our understanding of the cognitive processes underlying creativity in future studies.

## Supplementary Material

Supplementary Material

## Data Availability

All data needed to evaluate the conclusions in the paper are present in the paper and/or the Supplementary Material, or are available at https://osf.io/dx63r/?view_only=080ae2eebd4a4d2ea1eda328fedf8019. This study was not preregistered. Analyses were conducted using open software and toolboxes available online as described in the Methods section (SPM 12: https://www.fil.ion.ucl.ac.uk/spm/software/spm12/; AFNI: https://afni.nimh.nih.gov); Nilearn: https://nilearn.github.io/stable/index.html; TEDANA: https://tedana.readthedocs.io/en/stable/; CPM: https://www.nitrc.org/projects/bioimagesuite/; Network metrics computation: https://sites.google.com/site/bctnet/home). Scripts are available at https://osf.io/dx63r/?view_only=080ae2eebd4a4d2ea1eda328fedf8019.
