# Supplementary material for: Switching, fast and slow: Deciphering the dynamics of memory search, its brain connectivity patterns, and its role in creativity
Source: Imaging Neurosci (Camb). 2025 Nov 18;3:IMAG.a.1018. doi: 10.1162/IMAG.a.1018 (PMC12628016; doi:10.1162/IMAG.a.1018)
Supplement: Supplementary Material [file IMAG.a.1018_supp.docx]

Supplementary Material for

# Switching, fast and slow: Deciphering the dynamics of memory search, its brain connectivity patterns, and its role in creativity

Marcela Ovando-Tellez^1,2,3^ *, Lucie Vigreux^1^ *, Yoed N. Kenett^4^, Mathias Benedek^5^, Thomas T. Hills^6^, Benoit Beranger^7^, Alizée Lopez-Persem^1^, Victor Altmayer^1^, Theophile Bieth^1,8^ & Emmanuelle Volle^1^

* These authors contributed equally to this work

**Affiliation**

^1^ Sorbonne University, FrontLab at Paris Brain Institute (ICM), INSERM, CNRS, Paris, France

^2^ Brain Connectivity and Behaviour Laboratory, Paris, France.

^3^ Groupe d’imaginerie fonctionelle (GIN), Institut des maladies Neurodegeneratives (IMN) – UMR 5293, CNRS, Bordeaux, France

^4^ Faculty of Data and Decision Sciences, Technion – Israel Institute of Technology, Haifa, Israel

^5^ Institute of Psychology, University of Graz, Graz, Austria

^6^ Department of Psychology, University of Warwick, University Road, Coventry CV4 7AL, UK.

^7^ Sorbonne University, CENIR at Paris Brain Institute (ICM), INSERM, CNRS, Paris, France

^8^ Neurology department, Pitié-Salpêtrière Hospital, AP-HP, F-75013, Paris, France

**Corresponding authors**

Emmanuelle Volle ([emmavolle@gmail.com](mailto:emmavolle@gmail.com)) & Marcela Ovando-Tellez (marcela.ovandot@gmail.com)

**Keywords**

Creativity– semantic foraging – fluency – executive control- semantic memory- functional connectivity

## Supplementary Material – Table of Contents

[**Supplementary Figures**](#_Supplementary_Figures)

- [**Supplementary Figure S1.**](#SF1) Mean Inter-Response Time and Inter-Response Semantic Similarity for clustering and switching responses for each cue word ...……..…. **Page 4**
- [**Supplementary Figure S2**](#SF2)**.** Mean Inter-Response Time and Inter-Response Semantic Similarity for clustering and switching responses …………………………...…. **Page 5**
- [**Supplementary Figure S3**](#SF3)**.** Patterns of *IRSr* during optimal foraging ……….... **Page 6**
- [**Supplementary Figure S4**](#SF4)**.** Optimal semantic foraging during PolyFT …...….. **Page 7**

[**Supplementary Tables**](#_Supplementary_Tables)

- [**Supplementary Table S1**](#ST1)**.** Different meanings of the French ambiguous words used in the PolyFT in Study 1 ………………………………………………………..…. **Page 8**
- [**Supplementary Table S2**](#ST2)**.** Number of different meanings of the French ambiguous words used as cue in the PolyFT in Study 2 ………………………………...….. **Page 9**
- [**Supplementary Table S3**](#ST3)**.** Descriptive statistics for the mean *IRTr* of clustering and switching responses ………………………………………………………...…. **Page 11**
- [**Supplementary Table S4**](#ST4)**.** Statistical tests for *IRTr* for global switching ...…. **Page 12**
- [**Supplementary Table S5**](#ST5)**.** Statistical tests for *IRTr* in *Fast-Switching* and *Slow- Switching* ……………………………...........................................................…. **Page 13**
- [**Supplementary Table S6**](#ST6)**.** Descriptive statistics for the executive function and creativity tasks ……………………………………………………………...…. **Page 14**
- [**Supplementary Table S7**](#ST7)**.** Relationship between the frequency of fast and slow clustering and switching responses and executive function and creative abilities ……………………………............................................................................…. **Page 15**
- [**Supplementary Table S8**](#ST8)**.** Study 2 – Relationship between PolyFT Fluency and Originality and propensity to adhere to the MVT or to use MVT-deviant responses ……………………………………………………………………………....…. **Page 16**

[**Supplementary Methods**](#_Supplementary_Methods)

- [**Supplementary Methods S1**](#_Supplementary_Methods_S1:)**.** The relatedness judgment task ……………..…. **Page 17**
- [**Supplementary Methods S2**](#_Supplementary_Methods_S2:)**.** Cue words used in Study 1 and Study 2 ………. **Page 18**
- [**Supplementary Methods S3**](#_Supplementary_Methods_S3:)**.** Creativity tasks ………………………….....…. **Page 18**
- [**Supplementary Methods S4**](#_Supplementary_Methods_S4:)**.** Executive function tasks ……………….…...…. **Page 19**
- [**Supplementary Methods S5**](#_Supplementary_Methods_S5:)**.** MRI data acquisition and preprocessing …...…. **Page 20**

[**Supplementary Results**](#_Supplementary_Results)

- [**Supplementary Results S1**](#_Supplementary_Results_S1:)**.** Longer inter-response times between than within cluster transitions …………………………………………………………………...…. **Page 23**
- [**Supplementary Results S2**](#_Supplementary_Results_S2:)**.** Response transitions within a cluster are semantically more similar than between clusters …………………………………….…...…. **Page 24**
- [**Supplementary Results S3**](#_Supplementary_Results_S3:)**.** Participants retrieve more dissimilar concepts than average during switching …………………………………………………………....…. **Page 24**
- [**Supplementary Results S4**](#_Supplementary_Results_S4:)**.** Dynamic analysis of *IRT* according to the switch position ……………………………………………………………………………....…. **Page 25**
- [**Supplementary Results S5**](#_Supplementary_Results_S5:)**.** Reliability of *IRT* correction in Study 2 ……..…. **Page 25**
- [**Supplementary Results S6**](#_Supplementary_Results_S6:)**.** Relationship between the frequency of fast and slow clustering and switching and executive function and creative abilities …...…. **Page 26**
- [**Supplementary Results S7**](#_Supplementary_Results_S7:)**.** Relationship between PolyFT fluency and originality and propensity to adhere to the MVT or to use MVT-deviant responses …….....…. **Page 27**

### **Supplementary Figures**


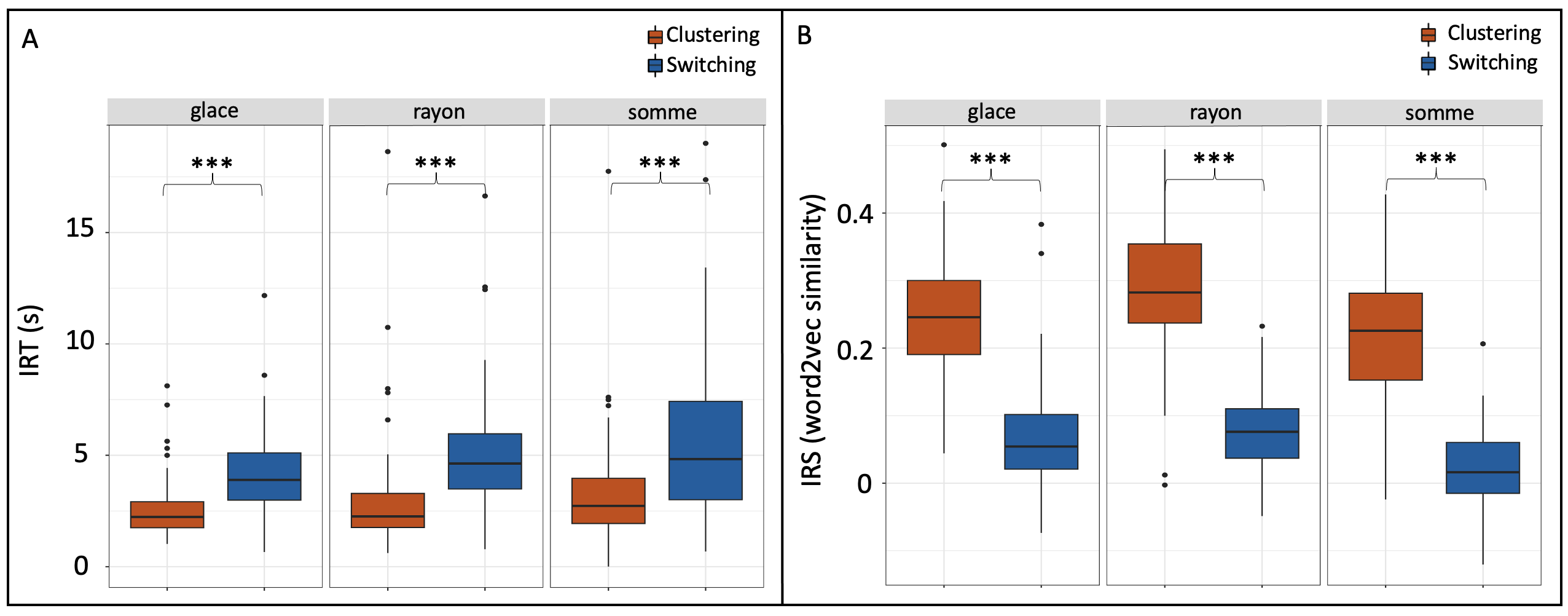


Supplementary Figure S1. **Mean Inter-Response Time and Inter-Response Semantic Similarity for clustering and switching responses for each cue word**. (**A**) The y-axis shows the mean *IRT* (seconds) for each color-coded process in the x-axis. (**B**) The y-axis shows the mean *IRS* (word2vec similarity) for each color-coded process in the x-axis. *** *p* < .001


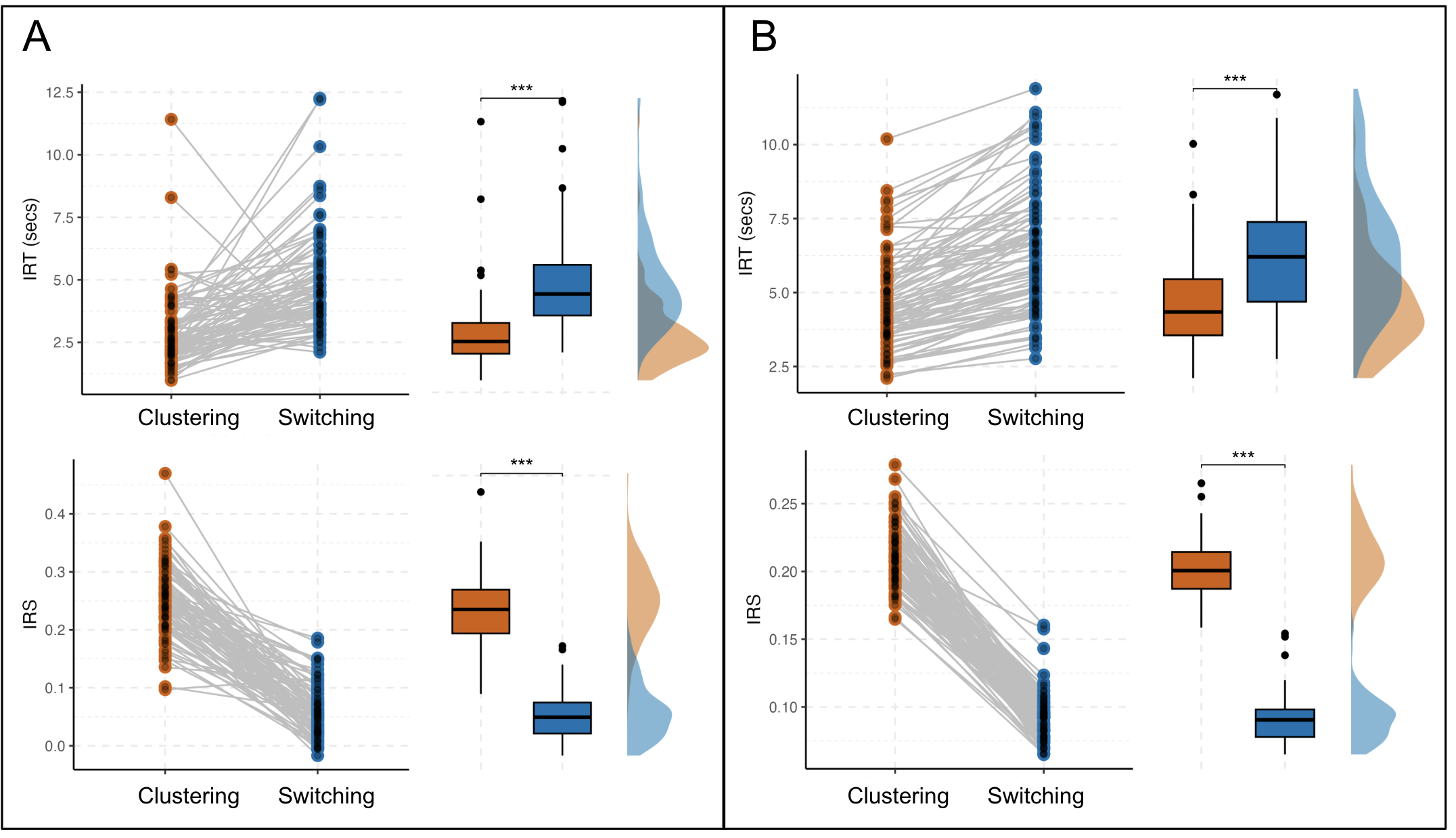


Supplementary Figure S2. **Mean Inter-Response Time and Inter-Response Semantic Similarity for clustering and switching responses**. Data are presented for the mean *IRT* (upper panels) and *IRS* (lower panels) for clustering and switching responses in Study 1 (**A**) and Study 2 (**B**). Each panel shows the data for each participant according to response type, barplots for the mean of the PolyFT cue words, and the distribution of responses. The y-axis shows the mean *IRT* (seconds) and mean *IRS* (word2vec similarity) for each color-coded response type. *** - *p* < .001


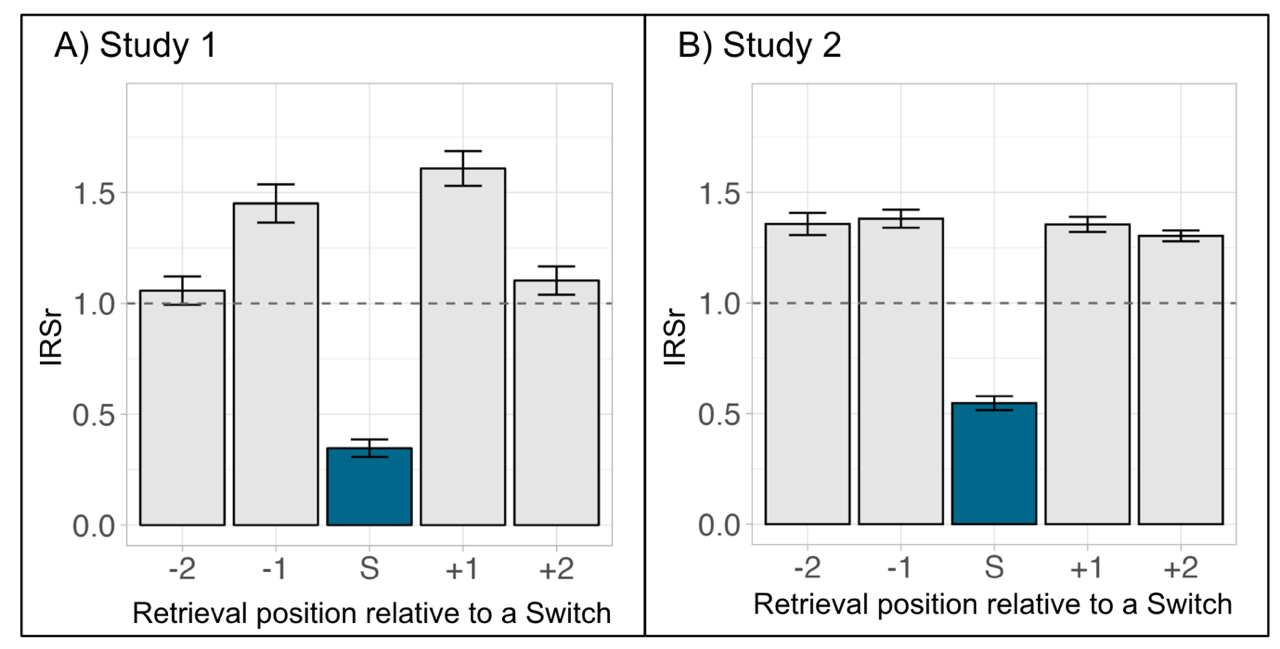


Supplementary Figure S3. **Patterns of *IRSr* during optimal foraging.** Values for *IRSr* are shown for each retrieval position in relation to switching for Study 1 (**A**) and 2 (**B**). The blue bars represent the values for the switching responses. The horizontal dashed line represents the *long-term IRS*. Error bars indicate the standard error of the mean.


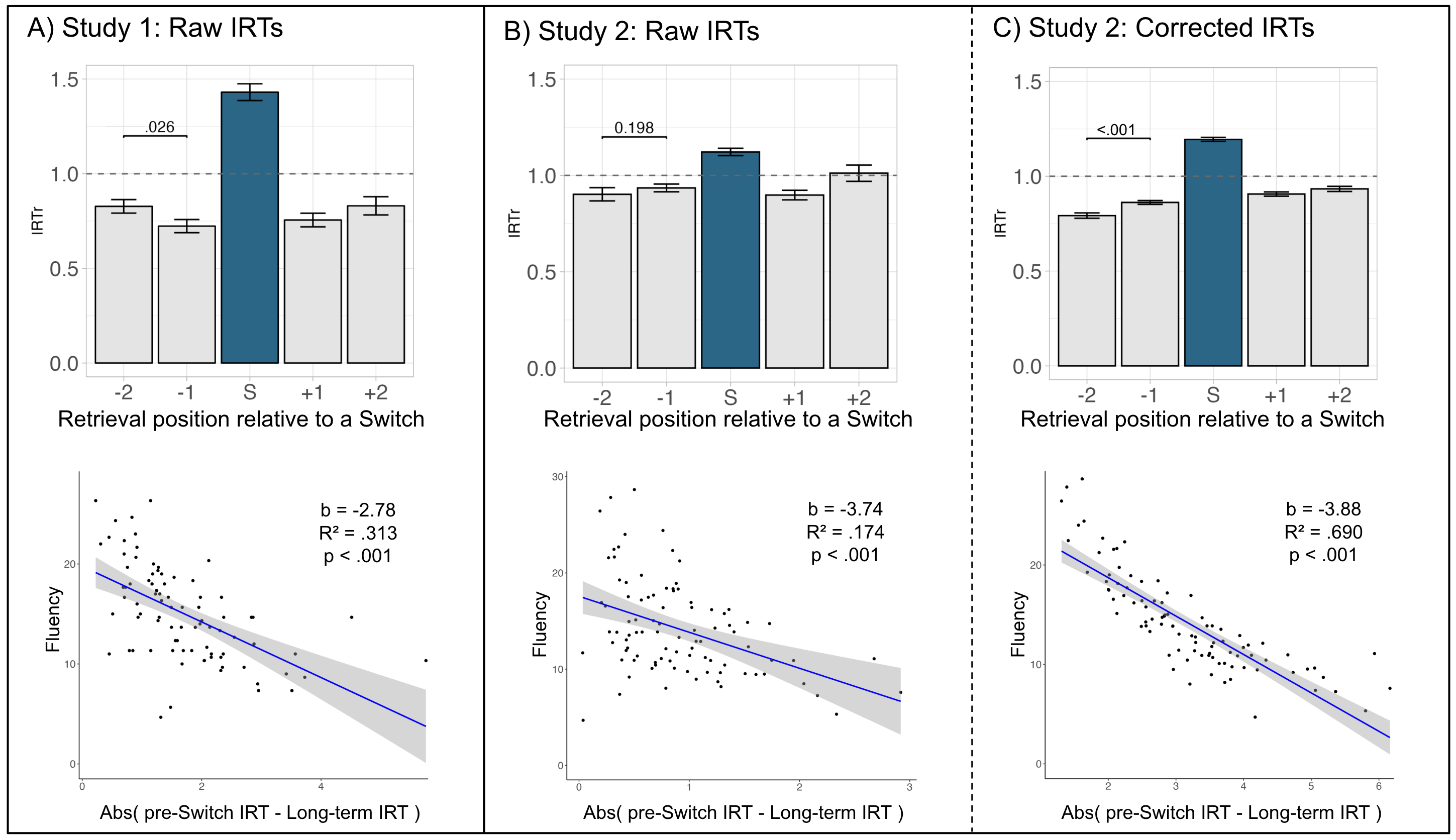


Supplementary Figure S4**. Optimal semantic foraging during PolyFT**. (Top) Values for *IRTr* are shown for each retrieval position in relation to switching for Study 1 (A) and Study 2 without (B) and with (C) *IRT* correction. The blue bar represents the values for the switching response. The horizontal dashed line represents the *long-term IRT*. Error bars indicate the standard error of the mean. (Bottom) Scatter plots showing the regression of the absolute difference between the *IRT* of the responses in the -1 position (i.e., pre-switch position) and the *long-term IRT* (x-axis) on the total number of responses retrieved for the whole PolyFT (y-axis) for Study 1 (A), 2 without (B) and 2 with (C) *IRT* correction.

### **Supplementary Tables**

Supplementary Table S1. **Different meanings of the French ambiguous words used in the PolyFT in Study 1.** The different meanings of the three cue words used in the PolyFT in Study 1 are presented separately in each column. Each word has at least five different meanings. In parenthesis is presented the total number of meanings for each cue word. A few words could not be categorized and were labeled as undetermined (< 2 % overall responses across participants)

| Somme (12) | Glace (5) | Rayon (12) |  |
| --- | --- | --- | --- |
| amount of money | ice | radius | |
| French region | ice cream | ray, beam |  |
| nap | mirror, window, looking-glass | radiation |  |
| to summon | emotional coldness | shelf |  |
| we are (verb to be) | icing or frosting | wheel spoke |  |
| work |  | honeycomb |  |
| summary (In sum) |  | domain |  |
| A mountain in France |  | range |  |
| lexical or phonetic proximity |  | diverge, spread |  |
| a river in France |  | joy |  |
| pack animal |  | light |  |
| sum (maths), amount, quantity |  | Department, row in a shop |  |

Supplementary Table S2**. Number of different meanings of the French ambiguous words used as cue in the PolyFT in Study 2.** Note that the cue word “indice” (in bold) was excluded from the analysis, as more than 25% of participants failed to produce a switch on that cue.

| Word in French | Number of meanings in French |
| --- | --- |
| aigu | 5 |
| appareil | 6 |
| baie | 4 |
| bande | 7 |
| base | 4 |
| boucle | 6 |
| cachet | 6 |
| canne | 7 |
| case | 7 |
| charge | 8 |
| classe | 5 |
| corps | 7 |
| côte | 5 |
| couche | 6 |
| cours | 10 |
| croissant | 3 |
| éclair | 3 |
| élan | 5 |
| essai | 5 |
| facteur | 3 |
| figure | 8 |
| forfait | 4 |
| glace | 5 |
| *indice* | ***4*** |
| ligne | 5 |
| mesure | 5 |
| mine | 10 |
| note | 5 |
| opération | 4 |
| ordre | 8 |
| partie | 4 |
| peine | 4 |
| pièce | 5 |
| pignon | 4 |
| pile | 7 |
| piste | 4 |
| plan | 9 |
| plat | 3 |
| plateau | 4 |
| point | 11 |
| rame | 6 |
| rayon | 12 |
| résolution | 4 |
| sens | 5 |
| signe | 6 |
| somme | 12 |
| terme | 3 |
| touche | 7 |
| trait | 7 |
| volume | 4 |

Supplementary Table S3. **Descriptive statistics for the mean *IRTr* of clustering and switching responses.** Then, mean and standard deviation (S.D.) are provided for global switching, fast switching, and slow switching responses, and the corresponding clustering for all positions for Study 1 and Study2.

|  | **Study 1** | | **Study 2** | |
| --- | --- | --- | --- | --- |
|  | mean | S.D. | mean | S.D. |
| **Global Switching** |  |  |  |  |
| *Clustering_IRTr* (position -2) | 0.83 | 0.33 | 0.79 | 0.14 |
| *Clustering_IRTr* (position -1) | 0.72 | 0.32 | 0.86 | 0.10 |
| *Switching_IRTr* | 1.43 | 0.41 | 1.20 | 0.10 |
| *Clustering_IRTr* (position +1) | 0.76 | 0.33 | 0.91 | 0.11 |
| *Clustering_IRTr* (position +2) | 0.83 | 0.45 | 0.93 | 0.13 |
|  |  |  |  |  |
| **Fast Switching** |  |  |  |  |
| *Clustering_IRTr* (position -2) | 0.67 | 0.41 | 0.80 | 0.18 |
| *Clustering_IRTr* (position -1) | 0.54 | 0.39 | 0.83 | 0.13 |
| *Switching_IRTr* | 0.53 | 0.14 | 0.53 | 0.04 |
| *Clustering_IRTr* (position +1) | 0.68 | 0.46 | 0.87 | 0.13 |
| *Clustering_IRTr* (position +2) | 0.71 | 0.53 | 0.91 | 0.17 |
|  |  |  |  |  |
| **Slow Switching** |  |  |  |  |
| *Clustering_IRTr* (position -2) | 0.95 | 0.48 | 0.79 | 0.15 |
| *Clustering_IRTr* (position -1) | 0.85 | 0.44 | 0.90 | 0.15 |
| *Switching_IRTr* | 2.22 | 0.55 | 2.07 | 0.21 |
| *Clustering_IRTr* (position +1) | 0.85 | 0.45 | 0.94 | 0.14 |
| *Clustering_IRTr* (position +2) | 0.95 | 0.60 | 0.95 | 0.20 |
|  |  |  |  |  |

Supplementary Table S4. **Statistical tests for *IRTr* for global switching*.*** Values represent the W value for Wilcoxon signed rank tests between each retrieval position in relation to a cluster switch (S). In bold are the correlations that remain significant after FDR correction for multiple comparison * *p* < .05; ** *p* < .01; *** *p* < .001

| Measure 1 |  | Measure 2 |  | Study 1 | Study 2 |
| --- | --- | --- | --- | --- | --- |
| Position -2 | - | Position -1 |  | 2336 * | **937 ***** |
|  | - | Position S |  | **164 ***** | **24 ***** |
|  | - | Position +1 |  | 2255 | **597***** |
|  | - | Position +2 |  | 1926 | **508***** |
|  |  |  |  |  |  |
| Position -1 | - | Position S |  | **149***** | **0 ***** |
|  | - | Position +1 |  | 1654 | **1279***** |
|  | - | Position +2 |  | 1532 | **1172***** |
|  |  |  |  |  |  |
| Position S | - | Position +1 |  | **3550 ***** | **4241***** |
|  | - | Position +2 |  | **3398 ***** | **4154 ***** |
|  |  |  |  |  |  |
| Position +1 | - | Position +2 |  | 1586 | 1863 |

|  |  |  |  |  |  |
| --- | --- | --- | --- | --- | --- |
|  |  |  |  |  |  |

Supplementary Table S5. **Statistical tests for *IRTr* in *Fast-Switching* and *Slow-Switching.*** Values represent the W value for Wilcoxon signed rank tests between each retrieval position in relation to a cluster switch (S). In bold are the correlations that remain significant after FDR correction for multiple comparison * *p* < .05; ** *p* < .01; *** *p* < .001

| *Fast-Switching* | | | | | |
| --- | --- | --- | --- | --- | --- |
| Measure 1 |  | Measure 2 |  | Study 1 | Study 2 |
| Position -2 | - | Position -1 |  | **1989 **** | 1740 |
|  | - | Position S |  | 1844 | **4209***** |
|  | - | Position +1 |  | 1417 | **1354**** |
|  | - | Position +2 |  | 1194 | **1014***** |
|  |  |  |  |  |  |
| Position -1 | - | Position S |  | 1544 | **4273***** |
|  | - | Position +1 |  | **950 **** | 1590 * |
|  | - | Position +2 |  | **926 *** | **1185 ***** |
|  |  |  |  |  |  |
| Position S | - | Position +1 |  | **1182 **** | **0 ***** |
|  | - | Position +2 |  | 1134 * | **3 ***** |
|  |  |  |  |  |  |
| Position +1 | - | Position +2 |  | 1594 | 1667 |
|  |  |  |  |  |  |
|  |  |  |  |  |  |
| *Slow-Switching* | | | | | |
| Measure 1 |  | Measure 2 |  | Study 1 | Study 2 |
| Position -2 | - | Position -1 |  | 1973 | **925***** |
|  | - | Position S |  | **49 ***** | **0 ***** |
|  | - | Position +1 |  | 2013 | **580***** |
|  | - | Position +2 |  | 1661 | **696***** |
|  |  |  |  |  |  |
| Position -1 | - | Position S |  | **13 ***** | **0 ***** |
|  | - | Position +1 |  | 1681 | **1476*** |
|  | - | Position +2 |  | 1230 | 1632* |
|  |  |  |  |  |  |
| Position S | - | Position +1 |  | **3462 ***** | **4278 ***** |
|  | - | Position +2 |  | **3090 ***** | **4278 ***** |
|  |  |  |  |  |  |
| Position +1 | - | Position +2 |  | 1327 | 2116 |

Supplementary Table S6. **Descriptive statistics for the executive function and creativity tasks**. The table reports the mean and standard deviation (S.D.) for all variables for the three executive function tasks: Digit span test (*Backward-span*), Trail Making Test (*TMT-shifting*) and Stroop test (*Stroop-interference*), and for creativity tasks: Combination of Associates Task (*CAT_CR, CAT_index* and *CAT_eureka*), and Alternative Uses Task (*AUT-fluency*, *AUT-ratings* and *AUT-uniqueness*)

|  | Study 1 | | Study 2 | |
| --- | --- | --- | --- | --- |
|  | mean | S.D. | mean | S.D. |
| *Backward-span* | 9.43 | 2.32 | - | - |
| *TMT-shifting* | 31.66 | 17.05 | 28.35 | 20.57 |
| *Stroop-interference* | 25.14 | 11.02 | - | - |
| *CAT_CR* | 0.47 | 0.12 | 0.50 | 0.12 |
| *CAT_index* | 0.42 | 0.23 | 0.58 | 0.29 |
| *CAT_eureka* | 0.69 | 0.20 | - | - |
| *AUT-fluency* | 19.88 | 8.35 | 19.47 | 8.94 |
| *AUT-ratings* | 1.71 | 0.43 | - | - |
| *AUT-uniqueness* | 7.44 | 4.98 | 7.29 | 4.71 |

Supplementary Table S7. **Relationship between the frequency of fast and slow clustering and switching responses and executive function and creative abilities*.*** Spearman correlation coefficients are presented. One-tailed t-test was used for significance testing in Study 2. In bold are the correlations that remain significant after FDR correction for multiple comparison. * *p* < .05; ** < .01; *** *p* < .001

| Study 1 | *Fast-Clustering* | *Fast-Switching* | *Slow-Clustering* | *Slow-Switching* |
| --- | --- | --- | --- | --- |
| *Backward-span* | .158 | -.065 | 5e10-4 | .261* |
| *TMT-shifting* | -.189 | -.125 | -.067 | -.232* |
| *Stroop-interference* | -.081 | -.107 | .028 | -.257* |
| *CAT_CR* | -.244* | .260* | -.154 | .092 |
| *CAT_index* | .105 | -.112 | .117 | .015 |
| *CAT_eureka* | .098 | -.123 | .160 | -.176 |
| *AUT-fluency* | **.455***** | .040 | **.315**** | .213* |
| *AUT-ratings* | .135 | -.014 | .037 | -.043 |
| *AUT-uniqueness* | **.381***** | -.096 | **.370***** | .048 |

| Study 2 | *Fast-Clustering* | *Fast-Switching* | *Slow-Clustering* | *Slow-Switching* |
| --- | --- | --- | --- | --- |
| *TMT-shifting* | .15 | .14 | .09 | **.23***** |
| *CAT_CR* | .17 | .14 | .11 | **.21*** |
| *CAT_ index* | -.11 | -.19* | -.13 | **-.23*** |
| *AUT-fluency* | **.36***** | **.39***** | **.39***** | **.36***** |
| *AUT-uniqueness* | **.32**** | **.33***** | **.34***** | **.35***** |
|  |  |  |  |  |

Supplementary Table S8. **Study 2 -** **Relationship between PolyFT Fluency and Originality and propensity to adhere to the MVT or to use MVT-deviant responses*.*** Linear mixed models were used to perform the analyses. PolyFT originality was assessed as one minus the mean response-to-cue semantic similarity. Adherence to the MVT was assessed using the absolute difference between *IRT* in position -1 (i.e., pre-switch position) and the *long-term IRT*. MVT-deviant responses were computed as a percentage of *Fast-Switching* and *Slow-Clustering*. For an easier interpretation of the results, this value was multiplied by minus one so that lower values indicate lower adherence to MVT.

| Outcome | Predictors | Beta | *p* value | Adjusted R^2^ model | F-statistic |
| --- | --- | --- | --- | --- | --- |
| PolyFT Fluency |  |  | <.001 | 0.70 | F(2,89) =106 |
|  | Intercept | 28.37 | <.001 |  |  |
|  | % of MVT-deviant | -14.87 | .067 |  |  |
|  | Adherence to MVT | 3.68 | <.001 |  |  |
| PolyFT Originality |  |  | <.001 | 0.32 | F(2,89) =22 |
|  | Intercept | 0.83 | <.001 |  |  |
|  | % of MVT-deviant | 0.11 | .003 |  |  |
|  | Adherence to MVT | 0.01 | <.001 |  |  |

### **Supplementary Methods**

### Supplementary Methods S1: The relatedness judgment task

Participants performed the relatedness judgment task inside the scanner during the MRI session. This task was developed to build the individual semantic networks in a previous study (Bernard et al., 2019; Ovando-Tellez, Kenett et al., 2022)*.* This task consisted of 595-word pairs that were all possible pairwise combinations of 35 words selected based on linguistic and semantic properties (see Bernard et al., 2019), for details about the method for selecting the words). For each trial, the pair of words was displayed on the screen with a visual scale ranging from 0 to 100 for four seconds. During the first two seconds (thinking time), participants were asked to think about the relatedness between the words. During the last two seconds (response time), a slider appeared on the screen, and participants could move it using a trackball to provide their response. Participants were instructed to judge how related the concepts were by using all the scale values and to validate their responses by clicking on the left button of the trackball within two seconds. The lower values of the scale indicate poorly related words (0 for unrelated words), while the higher values indicate strongly related words (100 for strongly related words). For each participant and each pair of words, we considered the relatedness rating as the value on the scale at the moment of the validation. When participants did not validate, we considered the value on the scale at the end of the two seconds as the relatedness judgment for the given word pair.

The task was divided into six runs of 100 trials (except for the 6th run with 95 trials). An inter-trial interval jittered from 0.3 to 0.7 seconds (mean 0.5; interval: 0.05) separated two successive trials. Each run comprised four blocks of 25 trials (except for the last block of the 6th run with 20 trials), separated by a 20-second cross fixation on the screen. Participants were presented with a 10-second cross-fixation at the beginning and end of each run. Between the runs, participants had a self-paced break in the scanner.

Before performing the relatedness judgment task, participants performed a motor and task training on a computer outside and inside the scanner. The motor training allowed participants to get familiar with the visual scale and the trackball. Within 25 trials, they were presented with a number and a visual scale ranging from 0 to 100. They were instructed to position the slider on the scale at the value of this number and to validate their response by pressing the left button of the trackball. After validation, they had visual feedback indicating the value of the position they had validated on the scale. The task training allowed participants to become familiar with the actual task. The parameters were the same as described for the relatedness judgment task, but we presented 15 trials to the participants with different words. For more details reading the training, see the Supplementary Material in Ovando-Tellez, Kenett et al., (2022).

### Supplementary Methods S2: Cue words used in Study 1 and Study 2

The PolyFT used ambiguous French words as cues. Participants were given the following instruction: *“I am going to tell you a word, and for this word I will ask you to give, for one minute, words that you associate with it or that are synonyms, words or ideas that the word makes you think of. Say your ideas as they come, trying to go as quickly as possible. Let your ideas flow, but the words you give must still be linked in some way to the proposed word. If the links are not obvious for some words, I will ask you at the end to explain why you proposed them. Do not give groups of words or proper nouns, just single words”.*

In Study 1, we selected three cue words, each with at least five different meanings. These words and their different meanings are presented in **Supplementary Table S1**. In Study 2, we extended the PolyFT to include 50 cue words with at least three different meanings (**Supplementary Table S2**). The new cues were selected following the same criteria as described for Study 1. We selected words with at least three different meanings according to the French linguistic resource for research (Centre National de Ressources Textuelles et Lexicales; <https://www.cnrtl.fr/>). The 50 cue words of the PolyFT were distributed across 4 blocks (12 trials for 2 blocks and 13 trials for two other blocks), with equivalent average lexical frequency and number of different meanings across cues between blocks. After data collection, we excluded the cue word “*indice*” from the analysis since more than 25% of participants did not switch for this cue.

### Supplementary Methods S3: Creativity tasks

### Alternative Uses Task (AUT)

Participants were given the following instructions: *“This task consists of finding alternative, unusual, and original uses for common objects. For example: “A brick is mainly used as a building material, but it could also serve…as a diving weight or as a cheap dumbbell…” You will be presented with three objects, one after the other. For each object, come up with as many alternative uses (original and/or unusual) as you can. You have a maximum of 3 minutes per object. Write down your answers, separating them with commas, and try to express your idea as clearly as possible. When you are done, if the time limit has not yet been reached, you may move on by telling the examiner, “I’m finished.” At the end of the 3 minutes, we will ask you to select the 2 ideas you consider the most creative and original, ranking them from most creative to least creative.”*

All participants' responses were cleaned (i.e., corrected for typo errors and homogenizing singular and plurals of the same idea). We calculated the *AUT_fluency* as the total number of unique responses generated by the participants. The *AUT_uniqueness* was quantified as the number of ideas generated by less than 5% of the participants. The database to compute the *AUT-uniqueness* included eight additional participants who performed the set of creativity tasks but not the PolyFT. The *AUT_ratings* were the evaluations provided by five experienced, external raters to the top 2 creative ideas (Benedek et al., 2013; Silvia et al., 2008). The raters were provided with written instructions translated into French from [https://osf.io/vie7s](https://protect.checkpoint.com/v2/___https://osf.io/vie7s___.YzJlOnRlY2huaW9uOmM6bzpiYzc4YTdkYjMwMGU4OGRiNjg0ODM1ODdjZWM5MDdlYjo2OmI4MTQ6OGEyMTE2NWVhZjk4YjA5NjhkOWU2YWVhYTAwYTg3ZDdiNWUyNTNjOTRlMTk5ODRmZTQ0MDE2MmE2MzQ5MDk5MzpwOlQ). They were asked to rate the creativity of the ideas using a Likert scale from 0 (not creative) to 4 (highly creative). The inter-rater reliability was good as evidenced by an intraclass correlation coefficient of .74. For *AUT-ratings*, scores were first averaged across raters for each object, then averaged across the three objects. All other AUT scores were computed as the sum of the scores for the three AUT objects.

### Combination of Associates Task (CAT)

For each trial, the three cue words were displayed on the screen, and participants were instructed to press the space bar immediately when the solution came to their minds and to write it down on the computer using the keyboard. Then, participants were asked to report if the solution was found with a feeling of insight or Eureka, and had five seconds to press the “v” key if they found the solution with an insight or the “n” key otherwise. We quantified the *CAT_CR* as the participants' accuracy in the task, computed as the percentage of total correct responses. We computed the *CAT_index* as the difference between the accuracy in the close and distant conditions, divided by the overall accuracy that reflects participants' ability to solve more distant trials when controlling for performance. We estimated the *CAT_eureka* score as the percentage of reported Eureka among correct trials. This score represents the ability of the participants to solve trials via insight versus a more analytical process.

### Supplementary Methods S4: Executive function tasks

### Digit span test

The digit span test of the Wechsler Adult Intelligence Scale (WAIS) comprises two parts, in which participants are given a string of numbers that increase in size and are asked to repeat them aloud. In the first part, participants were given 16 different strings of numbers going from 2 to 9 digits consecutively and were instructed to repeat them in the same order. In the second part, they were given 16 other strings from 2 to 8 digits and were instructed to repeat them reversely. Participants performed two trial examples before the actual tasks. We quantified the performance in the second part (*Backward-span*) by giving 1 point for each correct response. The sum of these points was used as a final score.

#### Trail-making test

The trail-making test comprises two parts. In the first part (A), participants were presented with numbers (1 to 25) distributed randomly on a paper. They were instructed to link the numbers in increasing order with a pen as fast as possible. In the second part (B), participants were presented with numbers (1 to 13) and letters (A to L) distributed randomly on a paper. They were instructed to alternate numbers and letters in increasing order as fast as possible. We quantified the difference between the time to complete the second minus the first part as *TMT-shifting*. Therefore, higher values reflect lower abilities in shifting.

#### Stroop test

The French version (Chatelois et al., 1993) of the Stroop test is composed of four parts. In the first part, participants were presented with 100 written names of different colors and were instructed to read them aloud. In the second part, participants were presented with 100 color squares and were instructed to name the color of the ink. In the third part, participants were presented with 100 written names of colors with different color ink and were instructed to name the color of the ink aloud. We recorded the time participants took to complete each part of the task. We quantified the interference effect (*Stroop-interference*) as the difference in time to complete the third (ink naming with interference) and the second part (color naming) (Chatelois et al., 1993).

### Supplementary Methods S5: MRI data acquisition and preprocessing

We collected whole-brain imaging on a 3T scanner (Siemens Prisma, Germany) with a 64-channel head coil while participants performed the relatedness judgment task (six runs). Data was collected using multi-echo echo-planar imaging (EPI) sequences. Each run consisted of 335 volumes acquired with a repetition time (TR) = 1600 ms, echo times (TE) for echo 1 = 15.2 ms, echo 2 = 37.17 ms and echo 3 = 59.14 ms, flip angle = 73°, 54 slices, slice thickness = 2.50 mm, isotropic voxel size 2.5 mm, Ipat acceleration factor = 2, multi-band = 3 and interleaved slice order. Next, we acquired a T1-weighted structural image using TR = 2300 ms, TE = 2.76 ms, flip angle = 9°, 192 sagittal slices with a 1mm thickness, isotropic voxel size 1 mm, Ipat acceleration factor = 2, and interleaved slice order. At the end of the session, we acquired resting-state data for 15 minutes (not used in this study) with the same characteristics as those described for the fMRI data. No volume was discarded from the fMRI data since the recording did not contain dummy scans. Functional volumes of each run were first despiked, slice timing corrected, and realigned to the first volume (computed on the first echo) using the afni_proc.py pipeline from the Analysis of Functional Neuroimages software (AFNI; https://afni.nimh.nih.gov). We then denoised the data using the TE-dependent analysis of multi-echo fMRI data (TEDANA; [https://tedana.readthedocs.io/en/stable/](https://protect.checkpoint.com/v2/___https://tedana.readthedocs.io/en/stable/___.YzJlOnRlY2huaW9uOmM6bzpiYzc4YTdkYjMwMGU4OGRiNjg0ODM1ODdjZWM5MDdlYjo2OjRiZDU6MzhjYWNkYmFiZWU4ZGFkMWVkMjcwOTY0ODFmMjA0ZjkwM2Y4YjRmNDM2M2ViMDI2NTdhN2ViMWY5MzdhMTZiYTpwOlQ)), version 0.0.9150. The TEDANA pipeline performs an optimal combination of the echo time series followed by a PCA to reduce the data and ICA to decompose the multi-echo BOLD data. The BOLD components were then classified as BOLD or non-BOLD, and the latter was removed, allowing the removal of thermal and physiological noise, including artifacts generated by motion, respiration, and cardiac activity. The last step included the co-registration of the denoised data on the T1-weighted structural image using the Statistical Parametric Mapping (SPM) 12 package running in MATLAB (R2017b, The MathWorks, Inc., USA) and normalization to the Montreal Neurological Institute (MNI) template. To spatially normalize the fMRI data, we used the transformation matrix computed from the normalization of the T1-weighted structural image using the default settings of the computational anatomy toolbox (CAT 12; [http://dbm.neuro.unijena.de/cat/](https://protect.checkpoint.com/v2/___http://dbm.neuro.unijena.de/cat/___.YzJlOnRlY2huaW9uOmM6bzpiYzc4YTdkYjMwMGU4OGRiNjg0ODM1ODdjZWM5MDdlYjo2OjFhOTg6NWQwYjMzZjZiMDljNDkyYjhhYjVlY2Y2Y2IyN2VlNzI3MmVkMTI4ZTAyNzEwNTczZDgwMjNmODc0ZmU2MzdiZjpwOlQ)) implemented in SPM 12. No participant was removed due to excessive head motion within a single run (> 2 mm translation or > 3° rotation), and no participant had mean Framewise Displacement > 0.5 mm (Power et al., 2014). The denoised and normalized fMRI data were entered in a general linear model in SPM to covary out the task-related signal from each run. We regressed out of the BOLD signal 24 motion parameters (standard motion parameters, first temporal derivatives, standard motion parameters squared, and first temporal derivatives squared) and the onsets and durations of each task-related event (reflection period, response period, inter-trial interval, cross-fixation periods, and change of the cross-fixation color). We then standardized and detrended the residuals of the GLM for each run and concatenated the residuals of the six runs, removing the between-runs rest periods. This approach follows the background connectivity approach (Cole et al., 2019), as intrinsic networks during task performance compared to rest facilitate the prediction of individual traits and differences in brain-behavior relationships (Cole et al., 2021; Greene et al., 2018; Jiang et al., 2020). These preprocessed data (i.e., concatenated residuals of the six relatedness judgment task runs) were used in the subsequent analyses.

### **Supplementary Results**

### Supplementary Results S1: Longer inter-response times between than within cluster transitions

To explore the processes occurring during semantic retrieval in light of the semantic foraging policies as in Hills et al. (2012), we first examined whether the *IRT* and the *IRS* differ between clustering and switching responses. For each participant and cue word, we averaged the *IRT* and *IRS*, respectively, for the responses classified as clustering (*Clustering_IRT* and *Clustering_IRS*) and switching (*Switching_IRT* and *Switching_IRS*). The *Switching_IRT* reflects the time it takes to move from one cluster to another, while *Clustering_IRT* reflects the retrieval time within the same cluster. The *Switching_IRS* reflects the semantic similarity between responses when moving from one cluster to another, while *Clustering_IRS* reflects the semantic similarity between responses when retrieving concepts within the same cluster.

In Study 1, we explored semantic retrieval patterns during the PolyFT in relation to the predictions of the marginal theorem value. The mean *IRT* across participants and cue words was 3.6 ± 1.4 s. We identified a mean of 3.7 ± 1.2 switches across participants, representing 28% of the mean retrieved associations, for a mean fluency of 15.1 ± 4.7 words. We first examined whether retrieving concepts during between-cluster (switching) rather than within-cluster (clustering) transitions takes more time. On a global average, the *Switching_IRT* was 4.87 ± 1.97 s, while the mean *Clustering_IRT* was 2.89 ± 1.49 s. The Wilcoxon test showed that *IRT* was lower when people stayed in a cluster (*Clustering_IRT*) than switched to a different cluster (*Switching_IRT*). This result was significant for the three cue words (cue1, glace: W = 351, *p* < .001; cue2, rayon: W = 461, *p* < .001; cue 3, somme: W = 542, *p* < .001; **Supplementary** **Figure S1A**), and for the mean of the three cue words (W = 312, *p* < .001; **Supplementary Figure S2A**).

We conducted the same analysis in Study 2 to test predictions from the MVT on an extended set of words. In this computerized version of the task, the mean *IRT* across participants and cue words was 5.47± 1.9 seconds. Additionally, we identified a mean of 5.2 ± 1.8 switches across participants for a mean fluency of 14.2 ± 4.9 words. Consistent with Study 1, the Wilcoxon paired sample test showed that *IRT* was lower for clustering responses than switching (*Clustering_IRT* = 4.64 ± 1.56 s, *Switching_IRT* = 6.397 ± 2.05 s. W = 4277, *p* <.001; **Supplementary** **Figure S2B**). Thus, as expected, both studies showed that it takes longer for participants to retrieve words between clusters than within clusters.

### Supplementary Results S2: Response transitions within a cluster are semantically more similar than between clusters

We verified that responses related to the same meaning were semantically closer than responses related to different meanings. In study 1, on average, the *Switching_IRS* was 0.06 ± 0.05, while the mean *Clustering_IRS* was 0.25 ± 0.06. The Wilcoxon test showed that *IRS* was lower when people switched to a different cluster (*Switching_IRS*) than when staying in a cluster (*Clustering_IRS*). This result was significant for the three cue words (cue1, glace: W = 3664, *p* < .001; cue2, rayon: W = 3357, *p* < .001; cue 3, somme: W = 2541, *p* < .001; **Supplementary** **Figure S1B**), and for the mean of the three cue words (W = 3740, *p* < .001; **Supplementary** **Figure S2A**).

These findings were replicated in the Study 2. The Wilcoxon paired sample test showed that IRS was significantly lower for between-clusters transitions (*Switching_IRS* = 0.092 ±0.02) than within-clusters transitions (*Clustering_IRS* = 0.21 ± 0.022, W = 4278, p < .001; **Supplementary** **Figure S2B**).

### Supplementary Results S3: Participants retrieve more dissimilar concepts than average during Switching

We explored whether a comparable pattern as the one observed for *IRTr* is observed for *IRSr*, but in the opposite direction, by testing if responses generated during within-cluster transitions are more similar to each other than the ones in between-cluster transitions. For each participant and each cue word, we calculated the *long-term IRS* as the mean word2vec similarity across responses. For each response, we calculated the *IRSr* as the ratio between each *IRS* and the *long-term IRS* for each of the cue words. Thus, values above or below 1 indicate higher or lower than average *long-term IRS*, respectively. We computed the *Clustering_IRSr* and *Switching_IRSr* as the mean *IRSr* in the corresponding responses.

In Study 1, we observed higher *IRSr* in responses during within-cluster transitions compared to between-cluster transitions. The *Switching_IRSr* (position S; **Figure S3A**) had a value of 0.35 ± 0.36, while *Clustering_IRSr* for responses before switching were higher (*Clustering_IRSr =* 1.06 ± 0.59 for position -2 and 1.45 ± 0.80 for position -1). *Clustering_IRSr* were also higher for responses after switching (*Clustering_IRSr =* 1.61 ± 0.73 and 1.10 ± 0.59 for positions +1 and +2, respectively). This pattern was also replicated in Study 2, where *Switching_IRSr* was lower than 1 (position S = 0.55 ± 0.31; **Figure S3B**), while *Clustering_IRSr* for responses before switching were higher than 1 (*Clustering_IRSr =* 1.36 ± 0.48 for position -2 and 1.38 ± 0.40 for position -1). *Clustering_IRSr* were also higher than 1 for responses after switching (*Clustering_IRSr =* 1.36 ± 0.33 and 1.30 ± 0.24 for positions +1 and +2, respectively).

### Supplementary Results S4: Dynamic analysis of *IRT* according to the switch position

We explored the dynamic of *IRT* before switching, the MVT predicting that *Clustering_IRTr* at position -1 (pre-switch) would be significantly closer to the *long-term IRT* than at position -2 (pre-switch). Study 1 did not confirm this prediction as *Clustering_IRTr* in position -2 was not different from position -1 (*p* = .026; non-significant after FDR correction). However, in Study 2, *Clustering_IRTr* in position -2 was significantly lower than in position -1 (*p* < .001). Thus, as expected and predicted by the MVT, participants switch to a new cluster when they get closer to their *long-term IRT*. All descriptive statistics for Study 1 and Study 2 are provided in **Supplementary Table S3**. The detailed results of the statistical tests for both studies are reported in **Supplementary Table S4**.

The detailed analysis of *IRTr* according to the switch position for fast switching and slow switching responses separately reinforces the difference between fast and slow switching. We found that *IRT* increases (or stays stable) from position -2 to position -1 before a slow switching response (as predicted by the MVT) whereas it decreases (or stays stable) before a fast switching response. All descriptive statistics for Study 1 and Study 2 are provided in **Supplementary Table S3**. The detailed results of the statistical tests for both studies are reported in **Supplementary Table S5**.

### Supplementary Results S5: Reliability of *IRT* correction in Study 2

In the computerized version of the PolyFT used in Study 2, participants typed their responses on the keyboard. *IRT*s, measured between the response validation and the beginning of the next response, were surprisingly short, and much shorter than in Study 1, where the responses were provided orally. This correction made *IRT*s more comparable to those observed in Study 1 (Study 1 mean *IRT*s = 3.6 ± 1.4 secs, Study 2 mean raw *IRT*s = .69 ± .58 secs and Study 2 mean corrected *IRT*s = 5.5 ± 1.9 secs). Note that longer mean *IRT*s were expected in Study 2 since the task duration was extended from 60 to 90 seconds, and *IRT*s typically increase as a function of time on task. We hypothesized that participants used the typing period (time between the start of typing a response and the press of the validation key) to anticipate their next response, rather than the inter-response period. We estimated participants’ typing speed to isolate participants’ writing time from cognitive processing time and reallocate the latter to the inter-response times.

Importantly, we re-analyzed hypothesis 1 to check whether responses followed the MVT using raw *IRT*s. As shown in **Supplementary Figure S4**, the main results from Study 1 were replicated using the raw *IRT*s, but reinforced using the corrected *IRT*s.

### Supplementary Results S6: Relationship between the frequency of fast and slow clustering and switching and executive function and creative abilities

We ran Spearman correlations between *Fast-Switching, Fast-Clustering, Slow-Clustering* and *Slow-Switching* and the different measures of creative abilities. In Study 1, for the CAT task, we found a significant negative correlation between *CAT_CR* and *Fast-Clustering* (*r_s_* = -.244, *p* = .024) but a positive correlation between *CAT_CR* and *Fast-Switching* (*r_s_* = .260, *p* = .015). No significant correlation was observed for *CAT_index* and *CAT_eureka.* For the AUT task, we found significant positive correlations between *AUT-fluency* and *Slow-Switching* (*r*_s_ = .213, *p* = .049), *Fast-Clustering* (*r_s_* = .455, *p* < .001) and *Slow-Clustering* (*r_s_* = .315, *p* = .003), and between *AUT-uniqueness* and *Fast-Clustering* (*r_s_* = .381, *p* < .001) and *Slow-Clustering* (*r_s_* = .370, *p* < .001). No significant correlation was found for *AUT-ratings*. The correlations between *AUT-fluency* and *AUT-uniqueness* and *Fast-Clustering* and *Slow-Clustering* remained significant after correction for multiple comparisons. Descriptive statistics for creativity and executive function tasks are presented in **Supplementary Table S6**. The Spearman correlation coefficients are presented in **Supplementary Table S7.**

Study 2 examined the correlations that were significant in Study 1. The performance in CAT measured by *CAT_index* correlated significantly with *Fast-Switching (r_s_= -.19, p = .037*) and *Slow-Switching (r_s_= -.23, p = .017*). *CAT_CR* correlated significantly with *Slow-Switching (r_s_= .21, p = .023*). Correlations between *CAT_index* and *Fast-Switching* did not survive after correction for multiple comparisons. Regarding divergent thinking abilities, we found significant correlations between *AUT-fluency* and *Slow-Switching* (*r*_s_ = .36, *p* < .001), *Fast-Clustering* (*r_s_* = .36, *p* < .001) and *Slow-Clustering* (*r_s_* = .39, *p* < .001), and between *AUT-uniqueness* and *Fast-Clustering* (*r_s_* = .32, *p* = .001) and *Slow-Clustering* (*r_s_* = .34, *p* < .001). All correlations remained significant after FDR correction. Descriptive statistics for creativity and executive function tasks are presented in **Supplementary Table S6**. The Spearman correlation coefficients are presented in **Supplementary Table S7.**

Overall, these results suggest that individuals with more fast clustering and slow clustering responses had higher divergent thinking abilities. In addition, our findings suggest that individuals with more fast switching have better associative combination abilities.

### Supplementary Results S7: Relationship between PolyFT fluency and originality and propensity to adhere to the MVT or to use MVT-deviant responses

In Study 2, we examined how MVT-consistent and MVT-deviant patterns related to the fluency and originality of the responses during PolyFT, which we used as marker of creative thinking during this task. To assess participants’ propensity for MVT- deviant behavior, we computed and summed the percentage of responses categorized as slow clustering and fast switching. For MVT-consistent behavior, we measured the absolute difference between IRT in position -1 (i.e., pre-switch position) and the *long-term IRT* (Hills et al., 2012). This value served as a measure of adherence to MVT*~~.~~* The originality of the responses was computed as the mean semantic similarity of each response to the cue. Semantic similarity was computed as described in the method section 2.1.4.2. This metrics was then averaged through cues. The fluency was the number of responses for each cue, averaged across cues.

We tested the implication of adherence to MVT and usage of MVT- deviant behaviors for originality and fluency within the PolyFT. We used separate linear mixed models with originality or fluency as dependent variables, and both the measures of MVT-consistent and MVT- deviant behavior as predictors. On the one hand, linear mixed models confirmed the predictions from MVT; PolyFT fluency was significantly related to participants’ adherence to MVT but not to the percentage of MVT- deviant behaviors (**Supplementary Table S8**). These results confirmed that fluency is not impacted by the propensity to engage into one or the other behavior but is solely predicted by adherence to MVT. On the other hand, both adherence to MVT and proportion of MVT- deviant behaviors positively contributed to PolyFT originality*.*

Overall, MVT consistent behavior were associated with a higher fluency and originality of responses. In contrast, an increased proportion of MVT- deviant behaviors was associated with more original responses. These results suggest that both MVT-consistent (fast clustering and slow switching responses) and MVT-deviant (fast switching and slow clustering responses) patterns play a role in retrieving original responses during PolyFT.

### **References**

Benedek, M., Mühlmann, C., Jauk, E., & Neubauer, A. C. (2013). Assessment of Divergent Thinking by means of the Subjective Top-Scoring Method: Effects of the Number of Top-Ideas and Time-on-Task on Reliability and Validity. *Psychology of Aesthetics, Creativity, and the Arts*, 7(4), 341–349. https://doi.org/10.1037/a0033644

Bernard, M., Kenett, Y., Ovando-Tellez, M., Benedek M. & Volle E., Building individual semantic networks and exploring thei relationships with creativity, *Proceedings of the 41st Annual Meeting of the Cognitive Science Society*, A. Goel, C. Seifert, C. Freksa, Eds. (Cognitive Science Society, 2019), pp. 128-144. <https://escholarship.org/uc/item/90z4k8b0>

Chatelois, J., Pineau, H., Belleville, S., Peretz, I., Lussier, I., Fontaine, F. S., & Renaseau-Leclerc, C. (1993). Batterie informatisée d’évaluation de la mémoire inspirée de l’approche cognitive. [A computerized memory test battery based on the cognitive approach.]. *Canadian Psychology/Psychologie Canadienne*, 34(1), 45–63. https://doi.org/10.1037/h0078803

Cole, M. W., Ito, T., Cocuzza, C., & Sanchez-Romero, R. (2021). The Functional Relevance of Task-State Functional Connectivity. *Journal of Neuroscience*, 41(12), 2684–2702. https://doi.org/10.1523/JNEUROSCI.1713-20.2021

Cole, M. W., Ito, T., Schultz, D., Mill, R., Chen, R., & Cocuzza, C. (2019). Task activations produce spurious but systematic inflation of task functional connectivity estimates. *NeuroImage*, 189, 1–18. https://doi.org/10.1016/j.neuroimage.2018.12.054

Greene, A. S., Gao, S., Scheinost, D., & Constable, R. T. (2018). Task-induced brain state manipulation improves prediction of individual traits. *Nature Communications*, 9(1), Article 1. <https://doi.org/10.1038/s41467-018-04920-3>

Hills, T. T., Jones, M. N., & Todd, P. M. (2012). Optimal foraging in semantic memory. *Psychological Review*, 119(2), 431–440. https://doi.org/10.1037/a0027373

Jiang, R., Zuo, N., Ford, J. M., Qi, S., Zhi, D., Zhuo, C., Xu, Y., Fu, Z., Bustillo, J., Turner, J. A., Calhoun, V. D., & Sui, J. (2020). Task-induced brain connectivity promotes the detection of individual differences in brain-behavior relationships. *NeuroImage*, 207, 116370. <https://doi.org/10.1016/j.neuroimage.2019.116370>

Ovando-Tellez, M., Kenett, Y. N., Benedek, M., Bernard, M., Belo, J., Beranger, B., Bieth, T., & Volle, E. (2022). Brain connectivity–based prediction of real-life creativity is mediated by semantic memory structure. *Science Advances*, 8(5), eabl4294. <https://doi.org/10.1126/sciadv.abl4294>

Power, J. D., Mitra, A., Laumann, T. O., Snyder, A. Z., Schlaggar, B. L., & Petersen, S. E. (2014). Methods to detect, characterize, and remove motion artifact in resting state fMRI. *NeuroImage*, 84. https://doi.org/10.1016/j.neuroimage.2013.08.048

Silvia, P. J., Winterstein, B. P., Willse, J. T., Barona, C. M., Cram, J. T., Hess, K. I., Martinez, J. L., & Richard, C. A. (2008). Assessing creativity with divergent thinking tasks: Exploring the reliability and validity of new subjective scoring methods. *Psychology of Aesthetics, Creativity, and the Arts*, 2(2), 68–85. <https://doi.org/10.1037/1931-3896.2.2.68>
